# Mechanism of Cationic Peptide-Induced Assembly of Gold Nanoparticles: Modulation of Electrostatic Repulsion

**DOI:** 10.1002/agt2.70043

**Published:** 2025-04-21

**Authors:** Benjamin Lam, Robert Ramji, Margaret Mullooly, Kristina D. Closser, Tod A. Pascal, Jesse V. Jokerst

**Affiliations:** 1 Aiiso Yufeng Li Family Department of Chemical and Nano Engineering, University of California, San Diego, La Jolla, California, USA; 2 Department of Chemistry and Biochemistry, California State University, Fresno, Fresno, California, USA; 3 Materials Science and Engineering Program, University of California, San Diego, La Jolla, California, USA; 4 Department of Radiology, University of California, San Diego, La Jolla, California, USA

**Keywords:** DLVO theory, gold nanoparticles, intermolecular interactions, peptides, reversible aggregation

## Abstract

The aggregation of plasmonic nanoparticles can lead to new and controllable properties useful for numerous applications. We recently showed the reversible aggregation of gold nanoparticles (AuNPs) via a small, cationic di-arginine peptide; however, the mechanism underlying this aggregation is not yet comprehensively understood. Here, we seek insights into the intermolecular interactions of cationic peptide-induced assembly of citrate-capped AuNPs by empirically measuring how peptide identity impacts AuNP aggregation. We examined the nanoscale interactions between the peptides and the AuNPs via UV-vis spectroscopy to determine the structure-function relationship of peptide length and charge on AuNP aggregation. Careful tuning of the sequence of the di-arginine peptide demonstrated that the mechanism of assembly is driven by a reduction in electrostatic repulsion. We show that acetylated N-terminals and carboxylic acid C-terminals decrease the effectiveness of the peptide in inducing AuNP aggregation. The increase in peptide size through the addition of glycine or proline units hinders aggregation and leads to less redshift. Arginine-based peptides were also found to be more effective in assembling the AuNPs than cysteine-based peptides of equivalent length. We also illustrate that aggregation is independent of peptide stereochemistry. Finally, we demonstrate the modulation of peptide-AuNP behavior through changes to the pH, salt concentration, and temperature. Notably, histidine-based and tyrosine-based peptides could reversibly aggregate the AuNPs in response to the pH.

## 1 ∣ Introduction

Peptide interactions with carbohydrates [1], lipids [2], nucleic acids [3], and proteins [4, 5] are fundamental to many biological processes. These interactions have been studied and engineered to develop diverse applications in diagnostics and therapeutics [6]. More recently, peptides have been utilized to tune the properties of nanomaterials, such as for increasing the circulation time of a drug [7], minimizing side effects by acting as a targeting moiety [8], or improving health outcomes as a therapeutic agent [9]. Peptides have also been used to aggregate nanoparticles and modify the chemical behavior of nanoparticles for desired outcomes. For instance, the aggregation of plasmonic nanoparticles can be utilized for colorimetric biosensing [10]. Likewise, many developing photothermal diagnostics and therapies rely on tuning nanoparticle aggregation to alter their optical and heating properties [11, 12].

Colloidal stability and, thus, strategies to control the aggregation of nanoparticles are often described by Derjaguin, Landau, Verwey, and Overbeek (DLVO) theory [13]. The principles of this theory suggest that nanoparticles remain colloidally stable when the van der Waals forces attracting the nanoparticles together are balanced by the electric double layer (EDL), repulsing the nanoparticles apart [14, 15]. These attractive and repulsive interactions are often summarized by the 12–6 or Lennard-Jones potential for point objects, with an energy minimum at the most favored interparticle distance [16-18]. The Derjaguin approximation extends interactions between molecular surfaces to calculate forces between three-dimensional bodies like nanoparticles [19]. The attractive forces are typically modeled by applying the Derjaguin approximation in combination with the material-specific Hamaker constants. Meanwhile, the repulsive EDL forces are modeled using expressions derived from the Poisson–Boltzmann equation, which describes the distribution of ions near charged surfaces, with the interaction strength determined by factors such as surface potential, ionic strength, and the resulting Debye length. However, this theory assumes that these are the primary forces exerted between nanoparticles and may neglect additional forces, such as hydrophobic interactions, hydrogen bonds, and specific ion effects [20, 21]. These limitations lead to challenges in precisely describing nanoscale behavior and leave the interaction between the aggregation-inducing agent and the nanoparticles unclear [22]. That is, a more thorough mechanism by which the aggregation-inducing components of the system (peptides, nucleic acids, polymers, salts, etc.) interact with the nanoparticles is needed to better understand and describe the phenomena beyond the framework of DLVO theory.

Our previous works have shown that citrate-capped gold nanoparticles (AuNPs) can be aggregated upon the addition of a di-arginine peptide [23, 24]. This aggregation produces a shift in the localized surface plasmon resonance (LSPR) of the particles and can be utilized in colorimetric biosensors for the detection of periodontitis, fungal diseases, and SARS-CoV-2 [25, 26]. More importantly, we have also shown that these aggregated nanoparticles can be dissociated upon addition of a thiolated polyethylene glycol polymer (HS-PEG) [27, 28]. In contrast, when the nanoparticles were aggregated through a reduction in the thickness of the EDL and charge screening by salt ions, subsequent dissociation with the HS-PEG was not possible [23]. This observation motivated us to more closely investigate the unique behavior of the peptides with the AuNPs during the aggregation. We reasoned that the mechanism of aggregation could proceed through linking the arginine ends between the AuNPs, forming an interconnected network of AuNPs cross-linked by the peptides. Alternatively, we also reasoned that the peptides could coat the surface of the AuNPs and cause aggregation by modulating their surface charge.

In this study, we aim to evaluate the mechanism by which cationic peptides interact with AuNPs to induce their aggregation. By doing so, we seek to develop and inform strategies for modulating the behavior between peptides and AuNPs. These fundamental insights into the intermolecular and surface forces may guide the development of biosensors and other nanoparticle-based technologies.

## 2 ∣ Experimental Section

### 2.1 ∣ Synthesis of Citrate-Capped AuNPs

Citrate-stabilized AuNPs (~20 nm) were prepared using the Turkevich method, as previously reported [23, 29]. Sodium citrate tribasic dihydrate (150 mg, 5 mL) was added to HAuCl_4_·3H_2_O (45 mg, 300 mL) under boiling conditions (120°C) and vigorous stirring. After 15 min, the solution mixture was cooled to room temperature. The resulting solution was centrifuged at 18,000 × *g* for 30 min. The supernatant was then discarded to remove unreacted reagents. The AuNPs were redispersed in 305 mL of deionized water and stored under ambient conditions.

### 2.2 ∣ Synthesis of Peptides

The peptides were synthesized through solid-phase peptide synthesis using an Aapptec Eclipse automated peptide synthesizer according to a previously reported procedure [25]. After synthesis, the resin was washed with *N*,*N*-dimethylformamide (12 mL DMF) and then dichloromethane (12 mL DCM). The resulting resin was dried under vacuum for at least 15 min. The peptide was cleaved from the resin using a cleavage cocktail consisting of phenol (300 mg), trifluoroacetic acid (5 mL), thioanisole (300 γL), deionized water (300 μL), and 2,2′-(ethylenedioxy) diethanethiol (150 μL). The resin was mixed with the cocktail under moderate shaking for at least 3 h. The cleaved peptide was then precipitated with 30 mL of −20°C diethyl ether and decanted after centrifugation at 9,000 RPM for 5 min. This step was repeated twice. Afterwards, the peptide was dried in a desiccator for 24 h prior to purification.

A list of the peptides used in this study is summarized in Table 1, and more details can be found in Table S1. All peptides in this study have a ─NH_2_ group for the N-terminal, a −CONH_2_ group for the C-terminal, and have the l-isomeric form of the amino acid unless otherwise noted. For acetylated N-terminal peptides, the resin was mixed with 4 mL DMF, 0.5 mL acetic anhydride, and 0.5 mL pyridine under moderate shaking for 30 min prior to the washing and cleavage steps as before. Wang resin was used for carboxylic acid C-terminal peptides. For the amide C-terminal peptides, the Rink-Amide MBHA resin was used (https://www.peptide.com/resources/peptide-synthesis-resins/).

### 2.3 ∣ High-Performance Liquid Chromatography Purification of Peptides

The dry peptide was purified according to a prior procedure [25]. In short, the peptide was dissolved in 3 mL of deionized water and 3 mL of acetonitrile. Afterward, the peptide was purified using a Shimadzu LC-40 High-Performance Liquid Chromatography (HPLC) system by monitoring the signal peak at 220 nm. The synthesis of the intended peptide was confirmed by verifying the mass of the peptide through electrospray ionization mass spectrometry (ESI-MS) using a Micromass Quattro Ultima mass spectrometer and Bruker Autoflex Max MALDI-TOFMS analysis. The ESI-MS/MALDI-TOF spectra are included in Figures S1-S7.

### 2.4 ∣ UV-Vis Spectroscopy

The UV-vis spectra of the AuNPs and the aggregated AuNPs were measured using a BioTek Synergy H1 microplate reader based on a previous procedure [23, 25]. In short, 100 μL of the AuNPs was added to 10 μL of the desired peptide in a 96-well plate. The UV-vis spectra were measured after 15 min. The assembly was characterized by measuring the ratio of the absorbance at the absorbance peak wavelength and at 520 nm. The resulting ratiometric assemblies were fitted to a sigmoidal curve, and the C_50_ was determined from the sigmoidal dose-response curve using GraphPad Prism 10.

### 2.5 ∣ Dynamic Light Scattering/Zeta Potential Measurements

The size and zeta potential of the AuNPs before and after aggregation were measured using a Zetasizer Nano ZS90 (Malvern Panalytical, Inc.). The AuNPs were diluted using deionized water. A minimum volume of 100 μL was used for the size measurements, and a minimum volume of 800 μL was used for the zeta potential measurements.

### 2.6 ∣ Multispectral Advanced Nanoparticle Tracking Analysis

The size of the AuNPs was measured using a Horiba Scientific Viewsizer 3000 via multispectral advanced nanoparticle tracking analysis (MANTA). This technique determines the particle size through tracking Brownian motion and the scattering of light from the particles using three lasers of wavelengths 635, 520, and 445 nm (e.g., red, green, and blue) [23, 30].

### 2.7 ∣ TEM Imaging

TEM images were acquired using a JEOL 1200 EX II. The TEM grid was prepared prior to image acquisition by placing the TEM grid (formvar/carbon 300 mesh Cu) ona10 μL drop of the sample. The resulting sample was allowed to dry at room temperature overnight.

### 2.8 ∣ Peptide Quantification

The amount of peptide remaining free in the solution was quantified using the Pierce Quantitative Fluorometric Peptide Assay (https://assets.fishersci.com/TFS-Assets/LSG/manuals/23290_quantpeptide_fluor_UG.pdf). In short, 10 μL of the sample was added to a black 96-well plate, followed by 70 μL of the assay buffer and 20 μL of the fluorescent dye. The fluorescence of the peptides was measured using an excitation wavelength of 390 nm and an emission wavelength of 475 nm after 5 minutes. The concentration of peptide remaining free in the solution was measured from the supernatant after centrifuging the aggregated AuNPs at 15,000 × *g* for 15 min. The peptide concentration of the remaining pellet was measured after redispersing the aggregated AuNPs with deionized water.

### 2.9 ∣ Effects of pH on Peptide-Induced Assembly

A Britton–Robinson buffer was prepared by mixing 0.04 M phosphoric acid, 0.04 M acetic acid, and 0.04 M boric acid according to a reported procedure [31]. The pH of the buffer was then adjusted using 0.2 M sodium hydroxide (NaOH). The Britton–Robinson buffer was added to the peptide prior to the addition of the AuNPs. The final pH of the solution was measured using an Apera Instruments LabSen 241–3SP pH probe. The pH probe was calibrated with Apera Instruments buffer solutions at pH 4, 7, and 10 prior to use. The absorbance was measured using a BioTek Synergy H1 microplate reader after 15 min and characterized as before. Final pH values of approximately 3, 7, and 12 were analyzed.

The reversible aggregation of the AuNPs through pH changes used 0.1 M HCl and 0.1 M NaOH to adjust the pH. The final pH of the solution was also measured using an Apera Instruments LabSen 241–3SP pH probe and was calibrated prior to use. The absorbance was measured as before.

### 2.10 ∣ Effects of Sodium Chloride on Peptide-Induced Assembly

Solutions of sodium chloride (NaCl) at various concentrations were prepared by dissolving NaCl in deionized water. The solutions were vigorously stirred to ensure that the NaCl was fully dissolved. The NaCl solution was added to the peptide prior to the addition of the AuNPs. Final working concentrations of 100, 50, 25, 10, 1, and 0.1 mM NaCl were analyzed. The absorbance was measured using a BioTek Synergy H1 microplate reader after 15 min and characterized as before.

### 2.11 ∣ Effects of Temperature on Peptide-Induced Assembly

An Eppendorf thermocycler was set to the desired temperature of 25°C, 37°C, or 60°C. The peptides and the AuNPs were then equilibrated to the desired temperature using the Eppendorf thermocycler for 30 min. Afterwards the AuNPs were rapidly added to the peptides and returned to the thermocycler to allow for aggregation for 15 min. The absorbance was then measured using a BioTek Synergy H1 microplate reader and characterized as before. The aggregation at 4°C was done similarly, except the peptides and the AuNPs were placed in a refrigerator set to 4°C.

### 2.12 ∣ Effects of Plasma on Peptide-Induced Assembly

The AuNPs were centrifuged at 15,000 × *g* for 15 min. The supernatant was discarded, and the AuNPs were redispersed in human plasma. Dose-response curves and absorbance measurements were conducted as before.

### 2.13 ∣ Computational Chemistry

The CREST program was used to sample peptide conformations with up to 6 kcal/mol above the lowest energy conformation [32]. Metadynamics were run using GFN-FF, and optimizations were computed at the GFN2-xTB level of theory with the GBSA implicit solvation model for water [33]. Zwitterionic forms of the peptides were used throughout. Conformations up to 1.5 kcal/mol above the lowest energy state from the CREST conformers were then further optimized using Q-Chem [34]. This was done as a two-step process using B3LYP-D3 [35] with the polarizable continuum model (PCM) [36] using the dielectric for water for solvation, first optimizing with the 6-31G* basis set, and then the lowest conformers were finally optimized using the def2-TZVP basis set.

## 3 ∣ Results and Discussion

**SCHEME 1 ∣:**
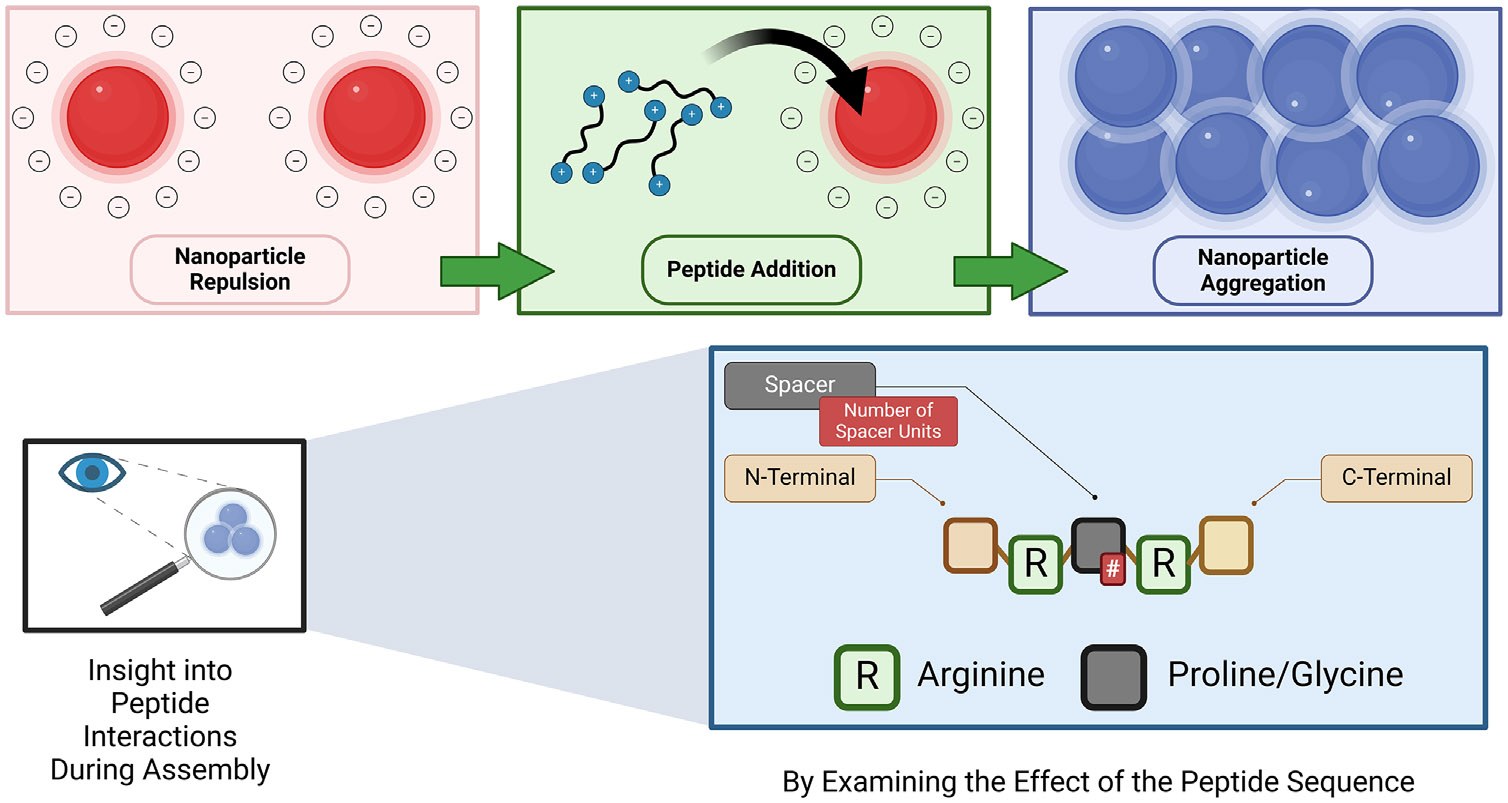
Strategy to experimentally probe the mechanism and orientation of peptide interaction during AuNP assembly. The nanoscale interactions between the peptides and the AuNPs were examined by evaluating the effect of changes in the peptide end terminals, size, stereochemistry, and solution environment.

The mechanism of aggregation between the peptides and the AuNPs was elucidated through a systematic investigation of the peptide sequence. Our results demonstrate that the aggregation mechanism proceeds primarily through the modulation of the AuNP surface charge. This work agrees with the findings of Tullman et al., who demonstrated that the AuNP assembly results from reduced electrostatic repulsion rather than “bridging” flocculation of the AuNPs [37]. In this study, the terms “assembly” and “aggregate” are used interchangeably. Scheme 1 highlights the experimental approach towards uncovering insights into this mechanism. More specifically, our investigation encompassed the following: (1) modification of peptide N- and C-*terminal groups* to probe end-group effects; (2) variation of *peptide length* through systematic addition of glycine or proline units; (3) examination of *stereochemical effects* via comparison of d- and l-arginine isomers; and (4) evaluation of *solvent effects* including pH, ionic strength, and temperature on peptide-AuNP aggregation behavior.

### 3.1 ∣ Di-Arginine Peptide-Induced Assembly

**FIGURE 1 ∣:**
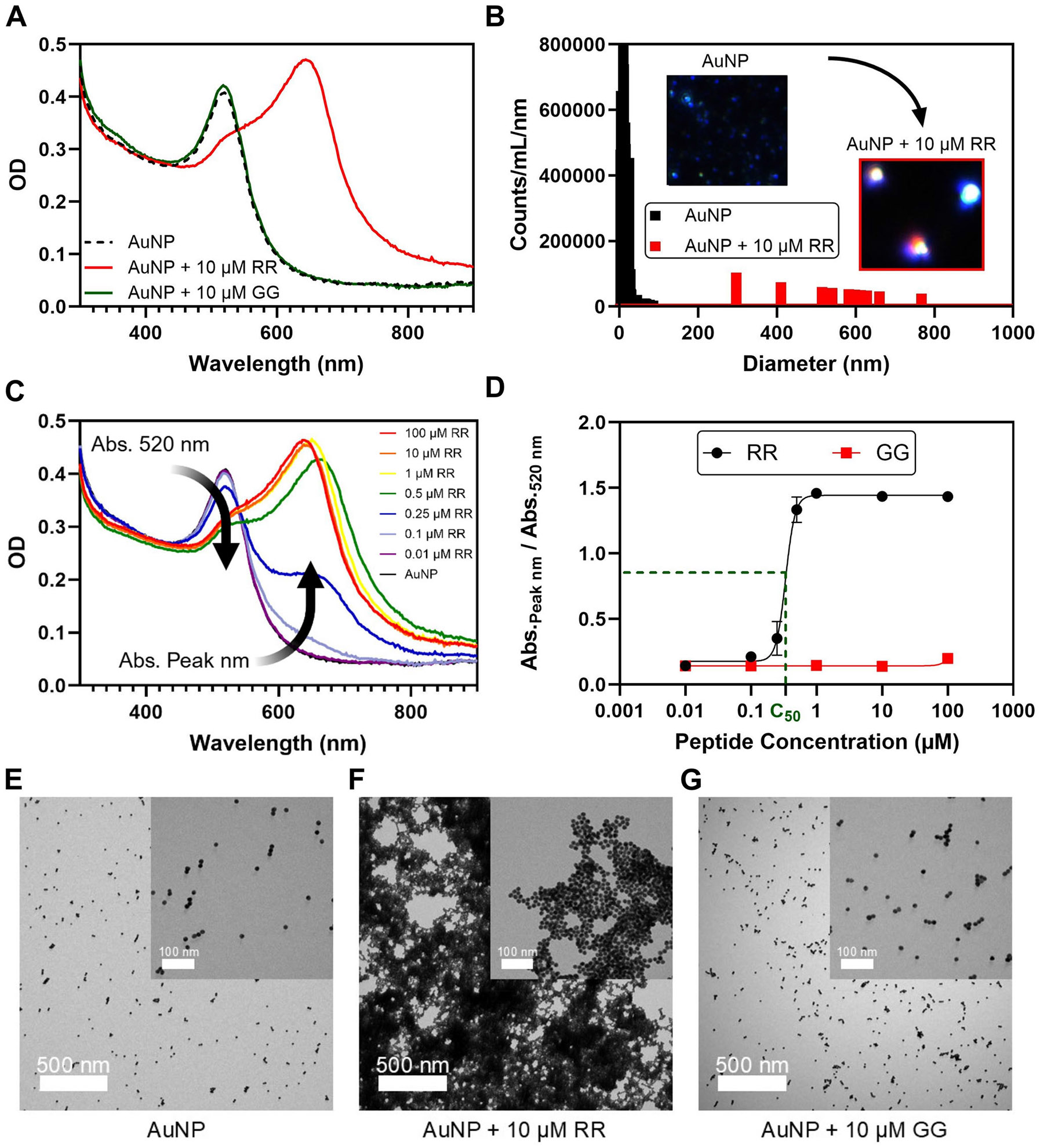
Characterization of di-arginine peptide-induced assembly. (A) UV-vis spectra illustrating the LSPR shift of the AuNPs upon aggregation with RR and the lack of spectral shift with the uncharged peptide GG. (B) NTA measurements confirming the increase in size of the nanoparticles upon aggregation with RR. Inset photos illustrate the shift from a blue light scattering of smaller-sized particles to the red and green light scattering of larger-sized particles. (C) Plot highlighting the spectral shift and the increase in the assembly intensity (Abs._Peak nm_/Abs._520 nm_) as a function of the RR concentration. (D) Dose-response curve highlighting the C_50_ concentration of RR needed to induce the AuNP aggregation. The C_50_ concentration represents the concentration at which the sigmoidal dose-response curve reaches 50% of its maximal value. Error bars represent the standard deviation of three replicates. TEM images confirm the aggregation of the AuNPs (E) upon the addition of the RR peptide (F) in contrast to the addition of the GG peptide (G).

Negatively charged citrate-capped AuNPs remain colloidally stable in solution due to electrostatic repulsion between the citrate coating of the nanoparticles [38]. However, upon the addition of the di-arginine peptide, the AuNPs spontaneously cluster together. This aggregation shifts the peak absorbance from a wavelength of 520–644 nm, resulting in a colorimetric change of the solution from red to blue (Figure 1A). This absorbance shift occurs rapidly within 15 min (Figure S8). The dipeptide Arg–Arg (RR) contains guanidinium groups from the arginine units with the positive charge delocalized among the nitrogen atoms (Figure S9) [39, 40]. The addition of the peptide Gly–Gly (GG), consisting of the neutral amino acid glycine, does not induce the spectral shift (Figure 1A). The diameter of the AuNPs increases in size from 18 nm to a cluster of approximately 500 nm after the addition of 10 μM RR (Figure 1B). The inset nanoparticle tracking analysis (NTA) images further confirm this size increase, revealing a shift from the blue light scattering of smaller particles to the red and green light scattering of larger particles [23]. Moreover, the particle count at 18 nm decreased by over 90%, indicating that most of the AuNPs were aggregated by the RR peptide. In contrast, the average size after the addition of 10 μM GG remained at approximately 20 nm (Figure S10).

The size of the aggregates depends on the concentration of RR (Figure S11). Figure 1C illustrates that the degree of spectral shift also depends on the peptide concentration. As such, the assembly intensity was quantified using the ratio between the absorbance at the peak wavelength after aggregation and the absorbance at 520 nm. A dynamic wavelength was chosen for the numerator because we reasoned that the absorbance peak could shift depending on the peptide used to aggregate the AuNPs. Therefore, the assembly intensity would be high when the nanoparticles are aggregated and low when the nanoparticles are dispersed. After fitting a sigmoidal dose-response curve to the assembly intensity values, the C_50_ value was defined as the concentration at which the assembly intensity reached 50% of its maximal value. A lower C_50_ would suggest that the peptide is more effective at aggregating the AuNPs, requiring fewer peptides to induce the absorbance shift. Figure 1D illustrates that RR has a C_50_ of 0.34 μM, while GG has an undefined C_50_ due to the lack of a strong absorbance shift even at concentrations of 100 μM.

The aggregation of the AuNPs was also verified by TEM imaging. Figure 1E illustrates the dispersed state of the AuNPs compared to the clustered state of the aggregated AuNPs in Figure 1F. Moreover, Figure 1G indicates the preserved dispersity of the AuNPs even after the addition of the GG peptide at 10 μM as was done with the RR peptide.

### 3.2 ∣ Effect of the Peptide Terminal Ends

**FIGURE 2 ∣:**
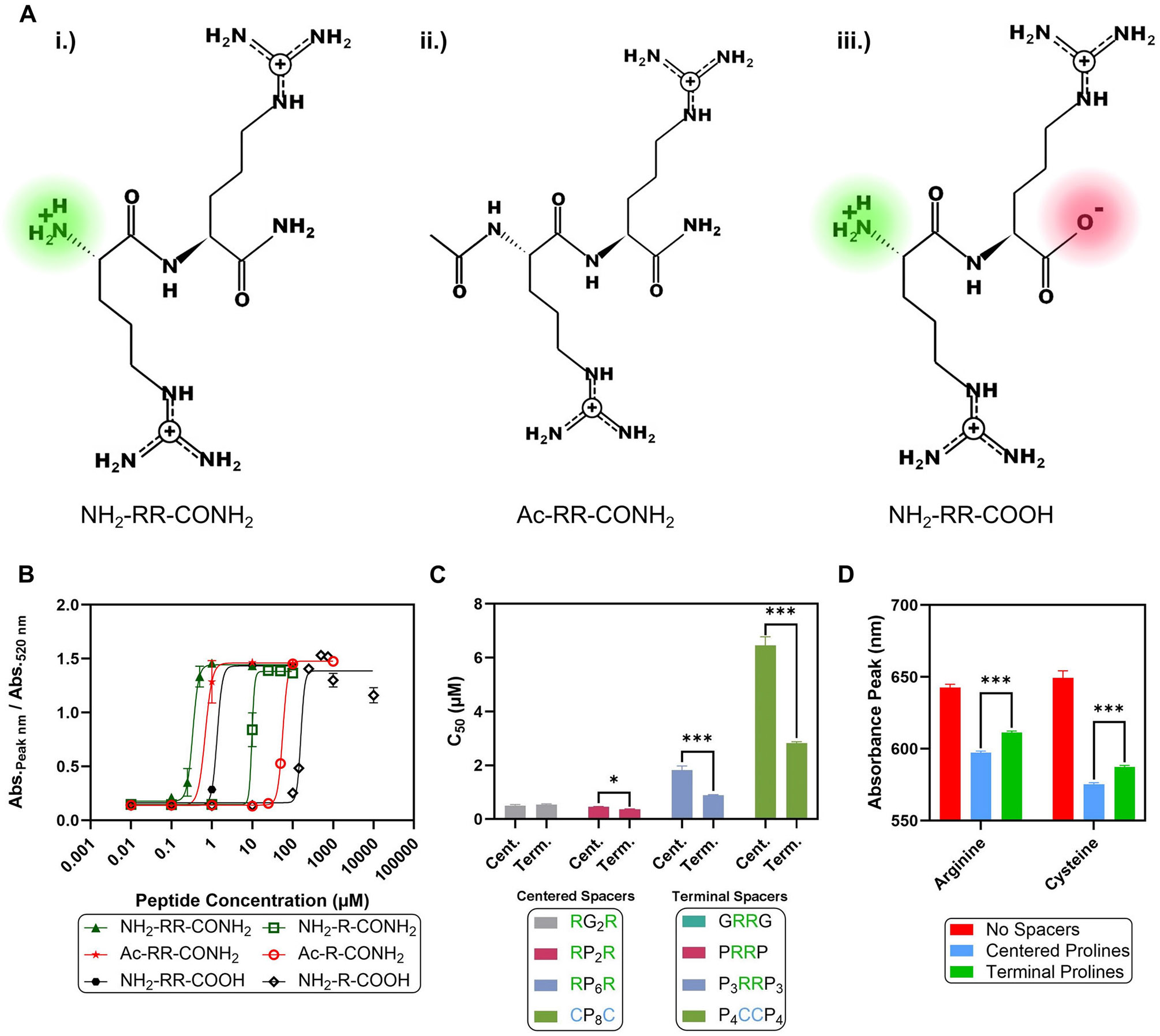
Effect of the terminal ends of the peptide. (A) Schematic highlighting the charge differences of the RR peptide with an acetylated N-terminal and carboxylic acid C-terminal. (B) Dose-response curves illustrate that a positive terminal group facilitates aggregation, while a negatively charged terminal hinders aggregation. This may be because of the electrostatic interaction of the negative terminal group with the negative surface charge of the AuNPs. (C) Bar plot illustrating that the peptide can still induce aggregation even when the spacer units are moved to the ends of the peptide. The peptides with the spacers on the terminal ends were more effective with a lower C_50_ concentration for both the di-arginine and di-cysteine peptides. (D) Bar plot comparing the UV-vis absorbance peaks of the peptides with and without proline spacers at different positions in the peptide. The LSPR peaks were redshifted with the spacers at the terminal ends. The above experiments were conducted at pH 7. All error bars represent the standard deviation of three replicates. Asterisks indicate statistical significance using Student’s *t*-test (*p < 0.05 and ***p < 0.001).

First, the effect of the peptide terminal ends during the aggregation process was investigated. We reasoned that the terminal group might play a lesser role in the aggregation process because the GG peptide had a positive N-terminal but still was unable to aggregate the AuNPs at 100 μM. An acetylated N-terminal and a carboxylic acid C-terminal were investigated (Figure 2A). The changes in the terminal groups result in differences in the global net charge and local charge distribution of the peptide [41-43]. The carboxylic acid C-terminal peptides (─COOH) are the expected natural form of the peptide, and amide-terminated peptides (−CONH_2_) are a non-natural variant that lacks the negative terminal end [43].

The propensity of the peptide to induce aggregation depends on its charge magnitude and localization (Figure 2B). The R peptides had an average C_50_ value of 75 μM (the average of the three terminal variants), while the RR peptides had an average C_50_ value of 0.8 μM. Therefore, the increase in the number of arginine units in the peptide resulted in almost 99% lower peptide concentrations needed to induce the aggregation. The C_50_ value decreases when adding additional arginine units along the inverse sixth power of the net charge in agreement with the Schulze–Hardy rule (Figures S12 and S13) [44, 45]. This suggests an electrostatic role in the aggregation mechanism because increasing the number of arginine units in the peptide results in a higher positive charge and increases its effectiveness in aggregating the AuNPs. Furthermore, the peptides with an amide C-terminal had lower C_50_ values compared to their respective counterparts with acetylation or a carboxylic acid group. This increase in aggregation ability likely arises from the additional positive charge from the N-terminal, which is similar to the effect of increasing the number of arginine units.

Although the acetylated N-terminal and carboxylic acid C-terminal peptides have the same net charge, the acetylated peptides were more effective in aggregating the AuNPs with a twofold reduction in their C_50_ values. This may be because the negative charge from the carboxylic acid group would hinder binding to the AuNPs due to electrostatic repulsion with the negatively charged citrate coating [46]. Computed minimum energy structures of NH_2_-R-COOH and NH_2_-RR-COOH show that the negative carboxylic acid in the dipeptide is fully stabilized by intramolecular hydrogen bonds, while for arginine alone, only one of the oxygens is stabilized intramolecularly (Figures S14-S16). These intramolecular forces may also contribute to the differences in their aggregation behavior. Interestingly, the Ac-RR-CONH_2_ and the NH_2_-RR-COOH peptides had 93% and 86% lower C_50_ values, respectively, compared to NH_2_-R-CONH_2_, even though all three peptides had the same net charge. This suggests that the greater flexibility of the side chain compared to the peptide backbone may enhance the accessibility of the positive charge, which would increase the ability of the peptide to induce aggregation. The delocalization of the positive charge on the guanidinium group compared to the single N-terminal nitrogen may also contribute to this difference.

We then evaluated whether the peptide would be more effective in inducing aggregation when the positive charge is placed in the center of the peptide rather than at the ends of the peptide. To investigate this question, the arginine units were spaced apart with the neutral amino acids glycine (G) or proline (P).

Figure 2C demonstrates that the peptides with terminal spacers had lower C_50_ values. This difference was magnified when the length of the peptide was increased with more spacer units. For instance, the PRRP peptide had an 18% lower C_50_ than the RPPR peptide, while the P_3_RRP_3_ had a 52% lower C_50_ than the RP_6_R peptide. However, the C_50_ values of RGGR and GRRG were comparable (*p* = 0.27). This may be because proline is bulkier than glycine, so the accessibility of the arginine unit between the spacers in RGGR compared to GRRG would not differ as much based on the placement of the spacers.

The sequence effect was also seen when the arginine units of the peptide were replaced with cysteines. The P_4_CCP_4_ had a 56% lower C_50_ than the CP_8_C peptide. Although cysteine is typically a neutral amino acid, cysteine can induce the aggregation of AuNPs through the formation of strong Au-S bonds and the displacement of the citrate ligand [47, 48]. As such, the surface charge of the AuNPs would become less negative due to the displaced citrates [42]. Therefore, the peptides with terminal spacers and central grafting points (PRRP, P_3_RRP_3_, and P_4_CCP_4_) may be more effective in inducing aggregation because they can more efficiently coat the surface of the AuNPs and alter the surface charge. The ability to aggregate the AuNPs with the arginine and cysteine units in the center of the peptide also supports an electrostatic mechanism for aggregation because the arginine or cysteine unit driving the aggregation does not have to be at the ends of the peptide to “link” the AuNPs together.

The absorbance peaks of the AuNP aggregates were also compared with different arrangements of the spacers. For both the arginine and cysteine peptides, the absorbance peaks were redshifted by 14 and 12 nm, respectively, when the spacers were at the terminal ends. This suggests that the AuNPs may be closer to one another with the terminal spacers because the LSPR peak experiences a bathochromic shift when the distance between the particles decreases [49].

### 3.3 ∣ Effect of the Peptide Size

**FIGURE 3 ∣:**
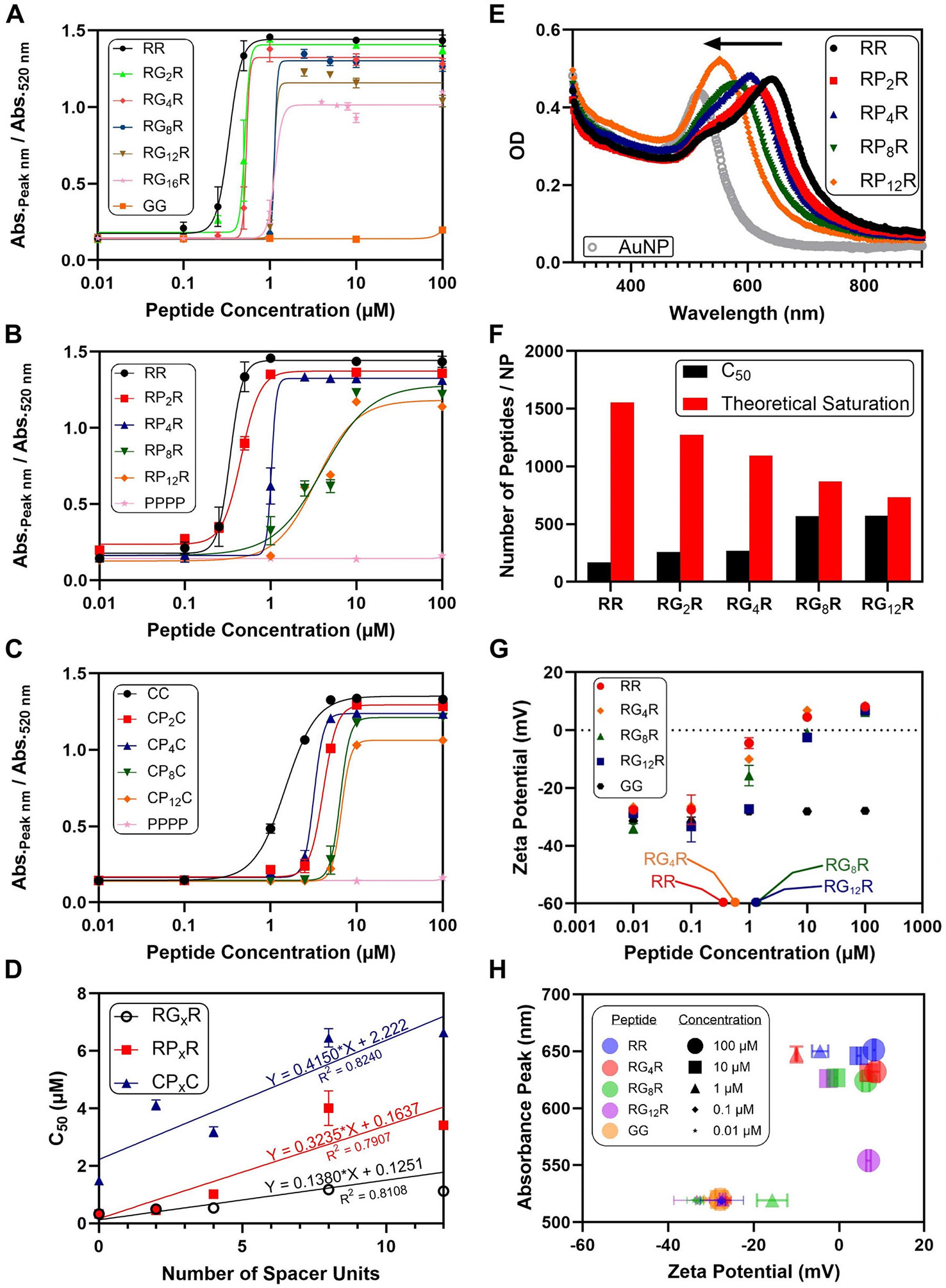
Effect of the peptide size. Dose-response curves illustrate the increase in peptide concentration required to induce AuNP aggregation as the peptide size increases for (A) glycine-spaced, (B) proline-spaced, and (C) cysteine-based peptides. (D) Linear regression analysis illustrating the greater increase in C_50_ concentration for the proline-spaced peptides RP_x_R and CP_x_C compared to the glycine-spaced peptides RG_x_R. (E) UV-vis spectra indicate greater redshift of the AuNPs for the smaller-sized peptides at 100 μM. (F) Bar plot comparing the peptide density on the AuNPs at their C_50_ concentration with a theoretical monolayer coverage. This suggests that the AuNP surface is not saturated upon the onset of aggregation. (G) Plot illustrating the zeta potential as a function of the peptide concentration. The C_50_ concentrations of the peptides are labeled on the *x*-axis. (H) Bubble plot illustrating the absorbance peak as a function of the zeta potential of the AuNPs for the RR, RG_4_R, RG_8_R, RG_12_ R, and GG peptides at different concentrations. The absorbance peak shift appears to occur roughly at −20 ± 8 mV. The above experiments were conducted at pH 7. All error bars represent the standard deviation of three replicates.

The effect of the peptide size on the aggregation of the AuNPs was investigated with lengths varying from 2 to 16 amino acid units. The size was tuned by varying the number of spacers in between the two arginine units. Figure 3A-C reveals that the sigmoidal dose-response curve shifts to the right as the number of spacers increases, indicating that a higher peptide concentration is needed to induce aggregation. As a negative control, the peptides GG and PPPP were used; both peptides were unable to produce a colorimetric response unless arginine or cysteine was added to the sequence.

Figure 3D highlights the increasing C_50_ values of the doseresponse curves as a function of the number of spacers. Based on the slope of the linear regression equations, the C_50_ values of the RP_x_R peptides and the CP_x_C peptides increased at a rate of 234% and 300% more per spacer unit than the RG_x_R peptides, respectively. This suggests that proline more effectively hinders the ability of the peptide to induce aggregation than glycine. This may be because proline is a bulkier amino acid consisting of a five-membered pyrrolidine ring compared to the single hydrogen atom of glycine [50]. This larger bulk may limit aggregation through increased steric hindrance and increased rigidity that would hinder the peptide from binding to the AuNPs. As a result, increased steric effects from the bulk of the spacer and the additional amino acid units would hinder adsorption, decreasing the surface charge modification and resulting in a higher C_50_. Moreover, the additional glycine and proline units would increase the length between the arginine units, leading to diminished charge density.

The slopes of both the RP_x_R and CP_x_C equations were significantly different than the slope of the RG_x_R equation (*p* = 0.01 and *p* < 0.0001, respectively); however, the difference in slope between the RP_x_R and CP_x_C equations was not statistically significant (*p* = 0.08). This suggests that the slope is more dependent on the identity of the spacer than on the identity of the “anchoring” amino acid. From the larger *y*-intercept values of the linear regression equations, the cysteine-based peptides were found to be less effective in aggregating the AuNPs than the argininebased peptides. The positive charge of arginine may make the arginine-based peptides more attracted to the negatively charged surface of the AuNPs compared to the neutral charge of cysteine. This binding of the positively charged arginine units compared to neutral cysteine units would lead to a more pronounced surface potential change, leading to AuNP aggregation at lower peptide concentrations.

The effect of the peptide size on the interparticle distance between the AuNPs was also considered. Figure 3E demonstrates the blueshift of the LSPR peak as the number of proline units increases in the peptide. The sizes of the aggregates were similar despite the increase in peptide length (*p* > 0.05) (Figure S17). The peak wavelength decreased by almost 100 nm when comparing the absorbance peak of RR to the absorbance peak of RP_12_R. This effect was also seen with an increase in the number of glycine units, but the effect was not as pronounced until 12 glycine units (Figure S18). This may be the result of increased steric hindrance from the larger peptides that would increase the separation distance between the AuNPs. However, the separation distance between the AuNPs did not seem to equal the length of the peptide. The RR peptide was estimated to have a length of 2 nm, while the RG_16_R peptide was estimated to have a length of 5 nm (Figure S19). Despite this, the AuNPs aggregated by RG_16_R appeared to have a separation distance of less than 5 nm, suggesting that the AuNPs are not directly linked across the linear length of the peptide (Figure S20). The aggregation mode induced by the RR and RG_16_R peptides also appeared to be diffusion-limited rather than reaction-limited. The fractal dimension (*d*_f_) was 1.7 for the RR-aggregated AuNPs and 1.8 for the RG_16_R-aggregated AuNPs (Figures S21 and S22). This is reflective of the more open structure of the clusters in diffusionlimited aggregation (*d*_f_ < 2) rather than the more compact clusters in reaction-limited cases (*d*_f_ > 2) [51].

The total nanoparticle surface area covered by the peptides was then considered as another potential factor in the aggregation mechanism of the AuNPs. In other words, we examined whether or not the aggregation was dependent on a certain amount of surface area coverage by the peptides. Assuming a spherical volume and taking the cross-sectional area as the peptide surface area, the larger peptides would naturally cover more of the nanoparticle surface area at a given concentration (Figures S23 and S24). The amount of peptide per AuNP at the C_50_ concentration was noticeably less than the theoretical saturation density (Figure 3F). This suggests that the aggregation mechanism hinges less on total surface area coverage and more on surface charge changes.

As the peptide binds to the AuNPs, the negative charge of the citrate-covered surface would be counteracted by the positive charge from the peptide. The binding of the peptide to the AuNPs leads to less peptide remaining free in the solution (Figure S25). For instance, 7.9 μM of RR remained free in the solution when 10 μM was added, suggesting that 2.1 μM was adsorbed to the AuNPs. This concentration corresponds to approximately 1050 RR/NP, which is below the theoretical monolayer coverage density of 1554 RR/NP. As more glycine units are added as spacers, the peptide density adsorbed onto the AuNPs increases by about 100 peptides per glycine spacer unit (Figures S26-S28). This may be because more of the larger-sized peptides are needed until the surface charge is neutralized, at which point the electrostatic driving force for further peptide binding is diminished. The change in the zeta potential of the AuNPs was determined to be dependent on the concentration and size of the peptide (Figure 3G). Moreover, there was a stronger increase in the zeta potential for the peptides with fewer spacer units and with terminal spacers (Figures S29-S31). For example, at 2 μM, the zeta potential of PPPRRPPP (C_50_ of 0.89 μM) was approximately 30% greater than the zeta potential of RP_6_R (C_50_ of 1.83 μM). This suggests that the peptides with a lower C_50_ were more effective in modifying the surface charge of the AuNPs, requiring less peptide per nanoparticle for a given potential change (Figure S32). Figure 3H illustrates the dependence of the absorbance peak on the zeta potential of the AuNPs. The absorbance peak redshifted only when the zeta potential was increased above approximately −15 mV. Some studies have suggested that nanoparticles are colloidally stable at zeta potential values below −30 mV and exceeding +30 mV [52, 53]. As such, the nanoparticles lose colloidal stability in between this range, supporting an electrostatically driven mechanism for aggregation of the AuNPs by the peptides. Furthermore, the zeta potential at the C_50_ values for the peptides was examined (Figure S33). The average zeta potential was −20 ± 8 mV, suggesting a threshold surface charge for aggregation of the AuNPs by the peptides in this range. Notably, the zeta potential remains negative at the C_50_ concentrations and becomes positive only at higher peptide densities.

The stereochemistry of the arginine unit also did not appear to have a substantial effect on the quantity of peptide required to induce aggregation of the AuNPs (Figure S34). The stereochemistry of the amino acid was evaluated to explore whether the conformational positioning of the arginine units could tune the assembly behavior. This suggests that the aggregation mechanism does not depend on a specific conformational structure of the peptide. In contrast, protease recognition of peptides often requires specific placement and binding of the substrate, leading to conformational selectivity [54, 55].

### 3.4 ∣ Effect of pH on the Assembly Mechanism

The effect of pH changes was evaluated to further confirm and tune the electrostatic interactions of the peptides with the AuNPs. We reasoned that changes to the pH would alter the peptide interactions with the AuNPs due to changes in the charge of the peptides from protonation or deprotonation. In particular, it was examined whether we could alter the ability of the peptide to induce or even reverse the aggregation of the AuNPs.

**FIGURE 4 ∣:**
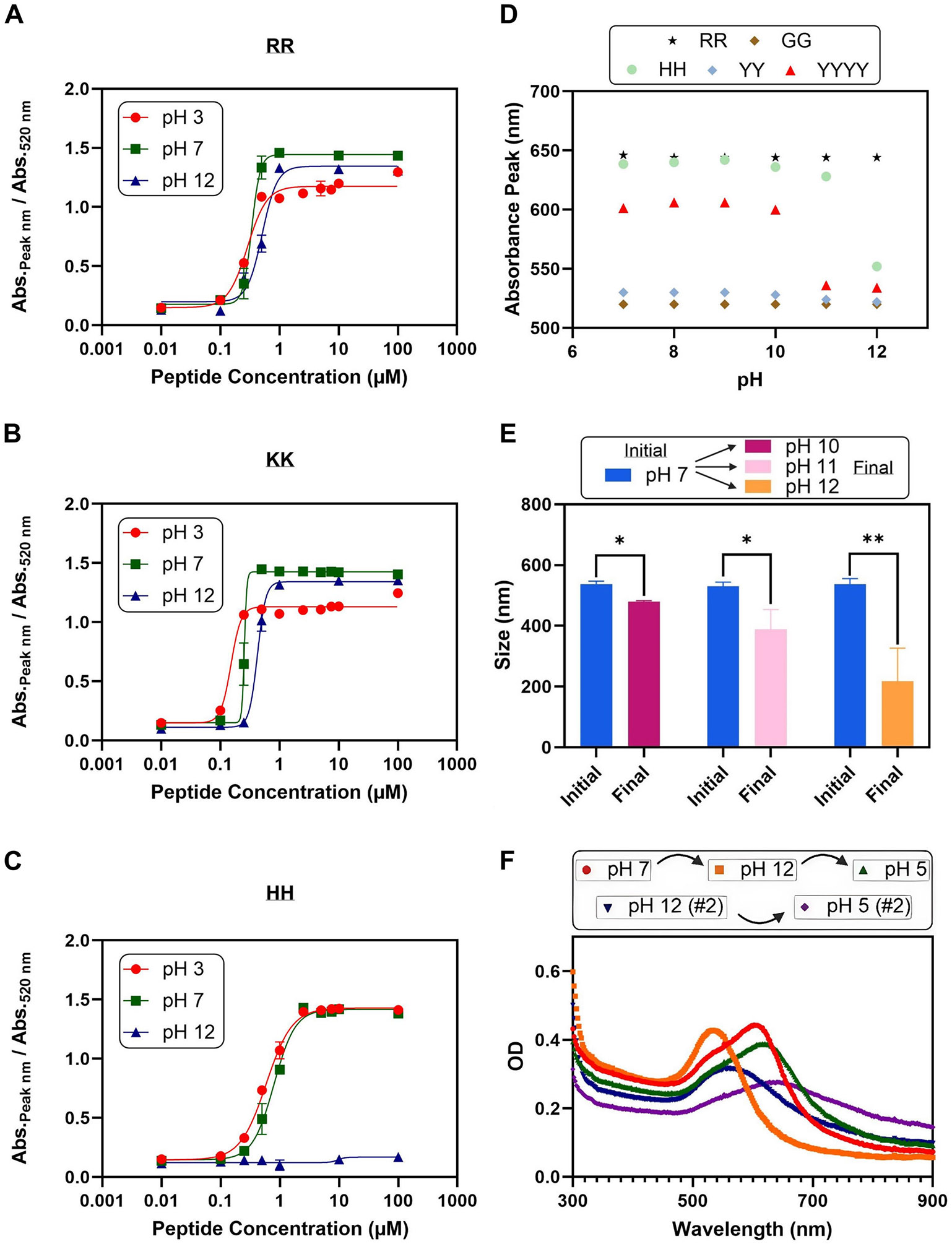
Effect of pH on the assembly mechanism. Dose-response curves for the peptides (A) RR, (B) KK, and (C) HH at pH 3, 7, and 12. At pH 3, the amount of peptide required to induce aggregation decreased, while it increased at pH 12. (D) Plot showing the absorbance peak of the AuNPs at increasing pH values. The LSPR peak for the peptides HH and YYYY was pH-responsive. (E) Bar chart indicating the size decrease of the YYYY-aggregated AuNPs at a pH of 10, 11, and 12 after increasing the pH from 7. (F) UV-vis spectra indicating the reversible cycling between aggregation and dissociation of the YYYY-aggregated AuNPs in response to pH changes. All error bars represent the standard deviation of three replicates. Asterisks indicate statistical significance using Student’s *t*-test (*p < 0.05 and ***p < 0.001).

Figure 4A-C shows the dose-response curves for the peptides RR, Lys–Lys (KK), and His–His (HH) at pH 3, 7, and 12. Dipeptides consisting of arginine, lysine, and histidine were chosen because these amino acids typically are cationic and possess a positive charge at pH 7 [56]. Changes in the pH beyond their p*K*_a_ would alter the protonation state of the amino acid and, consequently, the charge of the peptide (Figure S35). When the pH decreased from 7 to 3, the C_50_ value decreased by 15% for RR, 40% for KK, and 26% for HH. This indicates that the peptides were more effective at inducing AuNP aggregation at lower pH values. The decrease in pH likely results in more of the peptides existing in a protonated state, which would increase their affinity for binding to the AuNPs. This increased affinity would lead to more peptides binding to the AuNPs, resulting in a reduction in the electrostatic repulsion between the AuNPs that prevents their aggregation. However, the protonation of the citrate ligand (p*k*_a_ of 6.4) may also play a role in the greater susceptibility to aggregation due to diminished electrostatic repulsion between the AuNPs [57, 58]. Interestingly, the KK peptide had a lower C_50_ value compared to the RR peptide, even though both peptides had the same net charge. This may be because the delocalization of the positive charge over the guanidinium group for arginine may lead to diminished electrostatic interactions compared to lysine [59]. However, the effect of charge delocalization over larger volumes, such as throughout the length of an organic semiconductor, was not examined in this study [60].

Similarly, at a pH of 12, the C_50_ for RR increased by 51%, and the C_50_ for KK increased by 66% compared to the C_50_ value at pH 7. The HH peptide even loses its ability to aggregate the AuNPs at pH 12, producing minimal absorbance shifts even at a 100 μM concentration. This likely occurs because of the loss of the side chain’s cationic property from its deprotonation in a basic environment. While arginine and lysine have higher p*K*_a_s of 12 and 10 that allow the peptides to maintain their positive charge, histidine has a lower p*K*_a_ of 7, which makes it more susceptible to losing its positive charge [61-63]. The deprotonation of the arginine residue also appeared to have a greater effect than the deprotonation of the N-terminal amines (p*K*_a_ of 9) [41]. From a pH of 7–10, the C_50_ value increased by 0.06 μM, while from a pH of 10–12, the C_50_ value increased by 0.11 μM (Figure S36).

We then investigated whether the aggregated AuNPs could be dispersed electrostatically via pH changes. Figure 4D illustrates that the shift in the LSPR peak resulting from aggregation can be reversed upon increasing the pH. For instance, the HH-aggregated AuNPs had an absorbance peak of 640 nm at pH 7 but reverted to 552 nm upon increasing the pH to 12. However, the peptide RR was unable to be dissociated; this may be because the higher p*K*_a_ of arginine of 12 makes it more resistant to deprotonation. The peptide KK was also unable to be dissociated at pH 12 despite its slightly lower p*K*_a_ of 10 (Figure S37). The AuNPs were able to be dissociated by HS-PEG, though, for all three peptides (Figure S38).

The peptide Tyr-Tyr (YY) was then investigated since tyrosine has a lower p*K*_a_ value of 9 and would acquire a negative charge at pH values above its p*K*_a_ [64]. However, YY was unable to produce a substantial aggregation of the AuNPs, so YYYY was investigated instead. This tyrosine-based peptide likely aggregates the AuNPs through hydrophobic interactions and van der Waals forces because tyrosine is neutrally charged at pH 7 [63, 65]. Although the absorbance peaks blueshift, the aggregates are not completely dissociated. The absorbance peak only decreased to 534 nm for YYYY and 552 nm for HH compared to the peak wavelength of 520 nm for the pristine AuNPs. The dissociation of the aggregates for the YYYY peptide was confirmed through their decrease in size, as shown in Figure 4E.

The AuNPs were aggregated by the YYYY peptide at pH 7 after 5 min. Then, the pH was increased to pH 10, 11, and 12, and the sizes of the aggregates were remeasured. The deprotonation of tyrosine is supported by the decrease in the zeta potential of the aggregated AuNPs at higher pH values (Figure S39). The decrease in size aligns with the blueshift of the absorbance peak in Figure 4D. The average size of the aggregates decreased by over 50% at pH 12 to 218 nm, but the AuNPs were still larger than the original 18 nm size.

Figure 4F illustrates that the aggregation and dissociation of the AuNPs by the YYYY peptide can not only be reversed but also repeated. The aggregation and dissociation with the peptide HH also were reversible (Figure S40). The LSPR peak in the aggregated state seemed to gradually become broader and more redshifted compared to the original peak, with the peak shifting from 602 to 616 to 640 nm. Similarly, the LSPR peak in the dissociated state also gradually redshifted, increasing in wavelength from 520 to 532 to 552 nm. The redshift in the LSPR peak may be the result of the accumulation of H^+^ and Cl^−^ ions. This results in charge screening of the electrostatic forces and a reduction in the Debye length of the AuNPs [66].

### 3.5 ∣ Effect of NaCl on the Assembly Mechanism

**FIGURE 5 ∣:**
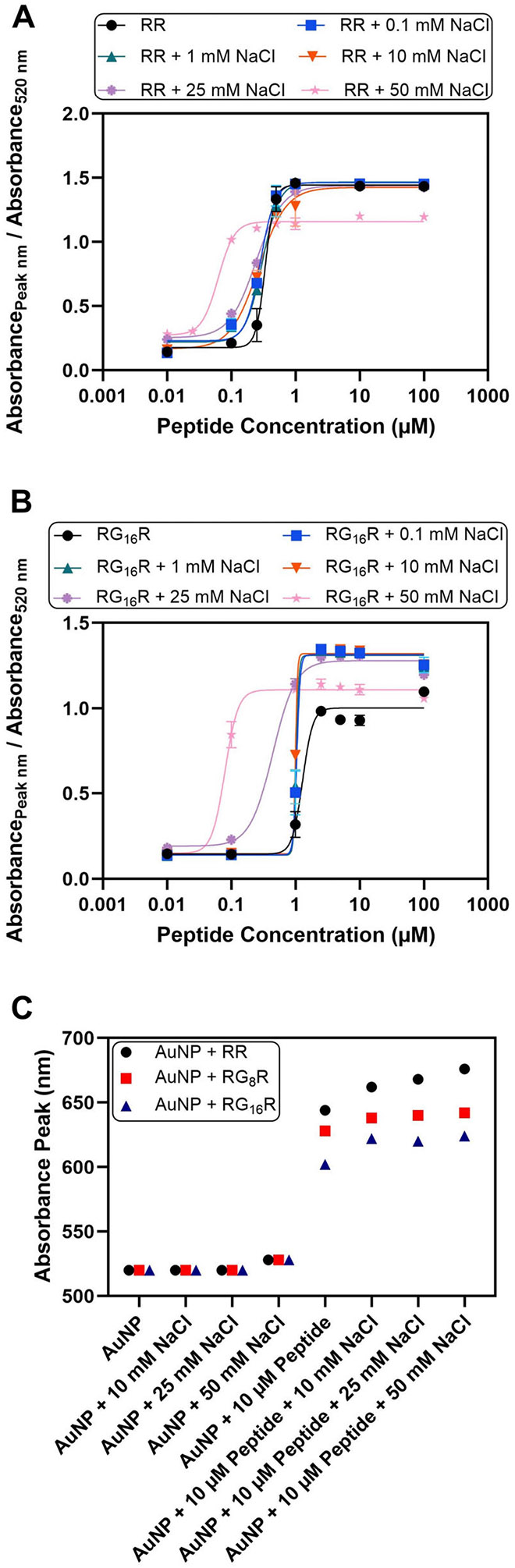
Effect of NaCl on the assembly mechanism. Dose-response curves illustrate lower peptide concentrations required to induce the AuNP aggregation in the presence of increasing NaCl concentrations for the (A) RR peptide and the (B) RG_16_R peptide. Error bars represent the standard deviation of three replicates. (C) Plot comparing the absorbance peaks for varying conditions of AuNPs, peptides, and NaCl concentrations. The LSPR shift is greater upon the addition of both NaCl and the peptide compared to the addition of only the peptide to the AuNPs.

The effect of NaCl on the peptide-induced assembly of the AuNPs was then investigated. Figure 5A,B illustrate that the C_50_ of RR and RG_16_ R decrease as the concentration of NaCl was increased from 0 to 50 mM. At concentrations exceeding 100 mM, the AuNPs were no longer colloidally stable due to diminished electrostatic repulsion and were aggregated even without the addition of any peptide (Figures S41 and S42). The decrease in C_50_ values was nonlinear, with the largest decrease in C_50_ values occurring at 25 and 50 mM (Figure S43). The Debye length ranged from 30.4 nm for 0.1 mM NaCl to 1.4 nm for 50 mM NaCl [67].

Figure 5C shows the absorbance peak with varying combinations of the AuNPs, peptides, and NaCl. The addition of only NaCl and no peptides to the AuNPs resulted in limited shifts to the absorbance peak; the peak remained in the 520 to 530 nm range without the addition of any peptides when the NaCl concentration was below 50 mM NaCl (Figure S44). However, the addition of the RR, RG_8_R, or RG_16_R peptides along with the NaCl resulted in a cooperative shift in the absorbance peak. That is, the absorbance peak shift was greater upon the addition of both the peptide and NaCl compared to the combined peak shift from adding each component separately. At 50 mM NaCl, the absorbance peak increased by 156 nm for RR, 122 nm for RG_8_R, and 104 nm for RG_16_R when both the peptide and NaCl were added simultaneously. When added separately, the absorbance peak only increased by 8 nm for 50 mM NaCl, 124 nm for RR, 108 nm for RG_8_R, and 82 nm for RG_16_R. Therefore, the redshift was approximately 10% greater than the additive effect. This increase in the absorbance peak is likely the result of charge screening of both the AuNPs and the peptides that allow the AuNPs to cluster closer together due to the diminished range of their electrostatic interactions.

### 3.6 ∣ Effect of Temperature on the Assembly Mechanism

**FIGURE 6 ∣:**
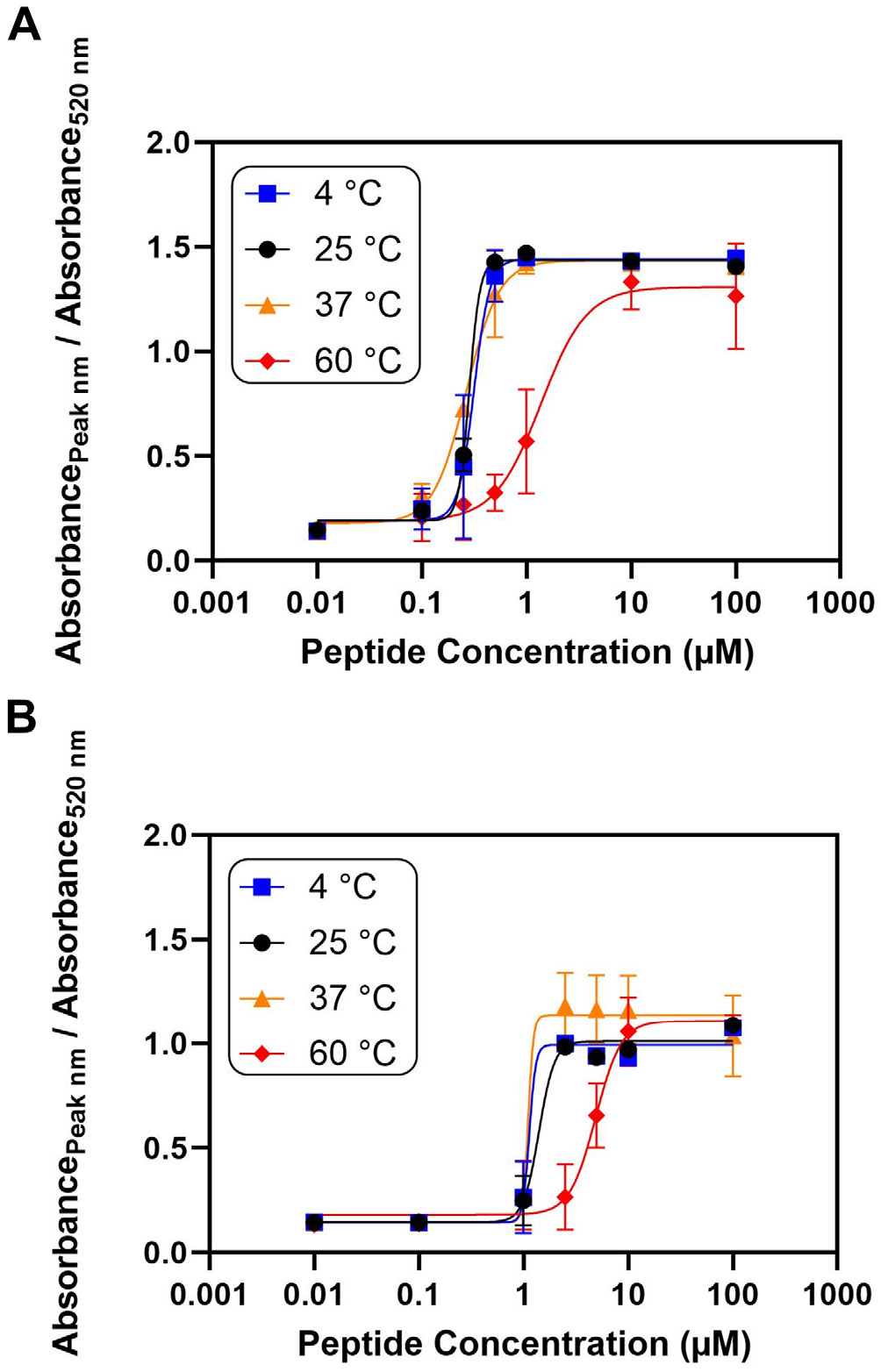
Effect of temperature on the assembly mechanism. Dose-response curves for the (A) RR peptide and the (B) RG_16_R peptide at temperatures of 4°C, 25°C, 37°C, and 60°C. The increase in temperature increased the concentration of peptide required to induce aggregation. Error bars represent the standard deviation of three replicates.

Finally, the effects of temperature on the assembly mechanism were investigated. We reasoned that increases in the temperature may hinder the aggregation of the AuNPs due to changes in the thermodynamics of the system and the increase in the kinetic energy of the nanoparticles. Figure 6A reveals that the C_50_ for RR increased from 0.3 to 1.4 μM upon increasing the temperature to 60° C. The sensitivity of the C_50_ to temperature was approximately 2%°C^−1^ (Figures S45 and S46) [68, 69]. Similarly, the C_50_ for RG_16_R increased from 1 to 3 μM, as shown in Figure 6B. This may be because the AuNPs would have greater kinetic energy at higher temperatures. This heightened energy state of the AuNPs may provide the particles with more energy to exit from a cluster of nanoparticles, limiting the aggregation of the AuNPs [70, 71]. Furthermore, the Gibbs free energy change from aggregation becomes less favorable at higher temperatures. From the Gibbs free energy equation, Δ*G* = Δ*H* – *T*Δ*S* (where Δ*G* is the change in Gibbs free energy, Δ*H* is the change in enthalpy, *T* is the temperature, and Δ*S* is the change in entropy), it can be seen that increases in the temperature increase the magnitude of the *T*Δ*S* term relative to the Δ*H* term [72]. Upon aggregation, the Δ*S* term would become negative due to the more “ordered” state of the nanoparticles. As such, at high temperatures, the Δ*G* term would increase, causing aggregation of the AuNPs by the peptides to be less thermodynamically favorable.

As the nanoparticles come together during aggregation, the peptides would also have less freedom of movement and become confined to the space in between the AuNPs. The limited space available would decrease the conformational entropy of the peptide and increase the change in the Gibbs free energy. This effect has been similarly examined with nanoparticles coated with long polymers becoming entropically stabilized due to steric hindrance between the polymer chains [67, 73].

### 3.7 ∣ Additional Forces and Factors

Even though electrostatic forces may serve as the primary factor governing the behavior between the cationic peptides and the AuNPs, additional intermolecular forces, such as hydrogen bonds and hydrophobic interactions, may also enhance or interfere with the assembly. For instance, hydrophobic interactions may facilitate AuNP aggregation by exerting an attractive force between hydrophobic regions of the peptides to minimize the exposed contact area with the solvent. These hydrophobic interactions can even lead to the aggregation of the AuNPs in peptide-free conditions; AuNPs suspended in Triton X-100 (a nonionic and amphiphilic surfactant) will spontaneously aggregate (Figure S47). On the other hand, hydrogen bonds and hydrophobic interactions may also lead to the formation of a protein corona around the AuNPs in complex biological media [74]. These proteins can interfere with the aggregation of the AuNPs with the peptides through steric and charge screening effects. For example, the presence of interfering compounds in pooled human plasma prevented the RR peptide from inducing aggregation. The RR peptide could only induce the absorbance shift when the plasma was diluted to below 0.05% (Figure S48). Despite this, prior work has shown that peptide-aggregated AuNPs could be redispersed upon the addition of HS-PEG in a wide range of biological media, including plasma, saliva, and urine [23]. As such, additional secondary forces may lead to deviations in the behavior between the peptides and the AuNPs in the presence of other molecules, such as multivalent ions and surfactants. These multivalent ions can aggregate the AuNPs in peptide-free conditions, resulting in a deep, primary energy minimum that prevents redispersion [23, 24]. Furthermore, differences in intramolecular forces may also alter the electrostatic behavior and stability of the peptide with the AuNPs (Figures S14-16).

While this study focuses on spherical AuNPs, different shapes, such as gold nanorods (AuNRs), may also exhibit different assembly behavior. Modulation of electrostatic interactions can lead to the aggregation of AuNRs [75, 76]. However, the geometry of AuNRs leads to facets that have different levels of energetic stability, which may subsequently affect peptide binding and aggregation orientation. The differences in the curvature of the sides and tips of the AuNRs would also affect the strength of the van der Waals forces between the AuNRs.

## 4 ∣ Conclusions

In summary, we have shown that the mechanism of cationic peptide-induced assembly of AuNPs appears to be primarily driven through a reduction in electrostatic repulsion rather than the linking of the AuNPs. The effectiveness of the peptides in inducing aggregation was found to be dependent on the peptide charge magnitude and distribution. Acetylated N-terminals and carboxylic acid C-terminals hindered aggregation, while greater charge density of arginine units enhanced aggregation. Increased size of the peptide with glycine or proline spacers impeded assembly, and arginine-based peptides were stronger at aggregating AuNPs than cysteine-based peptides at comparable lengths. The assembly mechanism was independent of amino acid stereochemistry but could be controlled through the effects of pH, salt concentration, and temperature. The interactions of the Na^+^ and Cl^−^ ions with the peptide produced a cooperative redshift in the absorbance, and histidine-based and tyrosine-based peptides were pH-responsive, enabling reversible aggregation.

Although the mechanism of aggregation appears to be governed by electrostatic interactions, further insights into the interaction between the peptides are the AuNPs have yet to be explored. For instance, the number of peptides bound to the AuNPs after dissociation has not been comprehensively quantified. Likewise, the location of peptide binding on the AuNPs (flat vs. curved surfaces) and the interactions with the HS-PEG could be explored. The aggregation of the AuNPs with other types of amino acids, such as with hydrophobic or polar amino acids, should also be evaluated. Comparisons between amino acids with similar properties (i.e., arginine and lysine) may also discern the effect of the amino acid identity. Future work should also utilize advancing computational methods to validate and predict the interactions of these and more complex peptide structures with the AuNPs. These insights will guide strategies for more precise control of peptide interactions not only for nanoparticles but also for many biological processes and other desired applications.

## Supplementary Material

si

Additional supporting information can be found online in the Supporting Information section.

## Figures and Tables

**TABLE 1 ∣:** Summary of Peptides.

Peptide	Rationale
RR	Positive control
GG	Negative control
Ac-RR-CONH_2_	Terminal group
NH_2_-RR-COOH	Terminal group
Ac-R-CONH_2_	Terminal group
NH_2_-R-COOH	Terminal group
R	Charge magnitude
RRR	Charge magnitude
RRRR	Charge magnitude
GRRG	Spacer distribution
PRRP	Spacer distribution
PPPRRPPP	Spacer distribution
RP_6_R	Spacer distribution
PPPP	Negative control
RG_2_R	Peptide size
RG_4_R	Peptide size
RG_8_R	Peptide size
RG_12_R	Peptide size
RG_16_R	Peptide size
RP_2_R	Peptide size
RP_4_R	Peptide size
RP_8_R	Peptide size
RP_12_R	Peptide size
CC	Peptide size
CP_2_C	Peptide size
CP_4_C	Peptide size
CP_8_C	Peptide size
CP_12_C	Peptide size
(D) NH_2_-R-COOH	Stereochemistry
(D) RR	Stereochemistry
HH	pH
KK	pH
YY	pH
YYYY	pH
