# Supplementary material for: Mechanism of Cationic Peptide-Induced Assembly of Gold Nanoparticles: Modulation of Electrostatic Repulsion
Source: Aggregate (Hoboken). Author manuscript; Available in PMC 2026 Apr 25. (PMC13108689; doi:10.1002/agt2.70043)
Supplement: si [file NIHMS2164036-supplement-si.docx]

**Supporting Information**

**Mechanism of Cationic Peptide-Induced Assembly of Gold Nanoparticles: Modulation of Electrostatic Repulsion**

Benjamin Lam^a^, Robert Ramji^a,c^, Margaret Mullooly^b^, Kristina D. Closser^b^, Tod A. Pascal^a,c^, and Jesse V. Jokerst^a,c,d*^

^a^ Aiiso Yufeng Li Family Department of Chemical and Nano Engineering, University of California, San Diego, La Jolla, CA 92093, United States

^b^ Department of Chemistry and Biochemistry, California State University, Fresno, Fresno, CA 93740, United States

^c^ Materials Science and Engineering Program, University of California, San Diego, La Jolla, CA 92093, United States

^d^ Department of Radiology, University of California, San Diego, La Jolla, CA 92093, United States

***Corresponding author’s email:** **[jjokerst@ucsd.edu (J.V.J.)](mailto:jjokerst@ucsd.edu (J.V.J.))**

Table of Contents

| **I. Materials** | **S3** |
| --- | --- |
| **II. Table of Peptides** | **S4** |
| **III. ESI-MS/MALDI-TOF Mass Spectra of Peptides** | **S6** |
| **IV. Size, Absorbance, and Computational Data** | **S9** |
| **V.** **Peptide Size and Nanoparticle Surface Area Coverage Calculations** | **S14** |
| **VI. Characterization of AuNP Assembly and Dissociation………………………..** | **S18** |
| **VII. References** | **S32** |

I. Materials

Trifluoroacetic acid (TFA, HPLC grade, >99%), piperidine (ReagentPlus®, 99%), and sodium citrate tribasic dihydrate (ACS Reagent, >99%) were purchased from Sigma Aldrich (St. Louis, MO). Thioanisole (>99%) and N, N-diisopropylethylamine (DIPEA, >99%) were purchased from Tokyo Chemical Industry Co., Ltd. (TCI). Fmoc-protected L/D-amino acids, hexafluorophosphate benzotriazole tetramethyl uronium (HBTU), Fmoc-Rink-Amide MBHA resin (0.67 mmol/g, 100-150 mesh), and Fmoc-Wang resin were purchased from AappTec, LLC (Louisville, KY). N, N-dimethylformamide (DMF, sequencing grade), acetonitrile (ACN, HPLC grade), ethyl ether (certified ACS), methylene chloride (DCM, certified ACS), acetic anhydride (certified ACS), pyridine (certified ACS), hydrochloric acid (HCl, certified ACS), sodium hydroxide (NaOH, certified ACS), boric acid (certified ACS), phosphoric acid (certified ACS), acetic acid (certified ACS), sodium chloride (NaCl, certified ACS) were from Fisher Scientific International, Inc. (Hampton, NH). L-R (NH_2_-R-COOH) and D-R (NH_2_-R-COOH) were also purchased from Fisher Scientific International, Inc. (Hampton, NH). Pierce Quantitative Fluorometric Peptide Assay was purchased from Thermo Fisher Scientific Inc. (Hampton, NH). Triton X-100 was purchased from Sigma Aldrich (St. Louis, MO). HS-PEG-OMe M.W. = 1,000 g/mol was purchased from Biopharma PEG (Watertown, MA). Pooled, human plasma was purchased from Sigma Aldrich (St. Louis, MO). Ultrapure water (18 MΩ·cm) was obtained from a Milli-Q Academic water purification system (Millipore Corp., Billerica, MA). TEM grids (formvar/carbon 300 mesh Cu) were purchased from Ted Pella (Redding, CA). The 96-well plates were purchased from BrandTech Scientific (Essex, CT).

II. Table of Peptides

Table S1. Table of peptides used in this study alongside their molecular weight, net charge, and rationale for their design. All peptides in this study have a -NH_2_ group for the N-terminal, a -CONH_2_ group for the C-terminal, and have the L-isomeric form of the amino acid unless otherwise noted. The molecular weights and net charge were determined using PepDraw. The carboxylic acid C-terminal peptides (-COOH) are the expected natural form of the peptide, and amide-terminated peptides (-CONH_2_) are a non-natural variant that lack the negative terminal end.

| Peptide | Molecular Weight (g/mol) | Net Charge | Rationale |
| --- | --- | --- | --- |
| RR | 329.2283 | +3 | Positive Control |
| GG | 131.0693 | +1 | Negative Control |
| Ac-RR-CONH_2_ | 371.2389 | +2 | Effect of Acetylation at N-Terminal |
| NH_2_-RR-COOH | 330.2124 | +2 | Effect of Carboxylic Acid Group at C-Terminal |
| Ac-R-CONH_2_ | 215.1380 | +2 | Effect of Acetylation at N-Terminal |
| NH_2_-R-COOH | 174.1115 | +1 | Effect of Carboxylic Acid Group at C-Terminal |
| R | 173.1274 | +2 | Effect of Single Arginine |
| RRR | 485.3292 | +4 | Effect of Additional Arginine Units |
| RRRR | 641.4301 | +5 | Effect of Additional Arginine Units |
| GRRG | 443.2711 | +3 | Effect of Arginine Placement Away from Terminal Ends |
| PRRP | 523.3335 | +3 | Effect of Arginine Placement Away from Terminal Ends with Bulkier Spacer |
| PPPRRPPP | 911.5439 | +3 | Effect of Placement of Arginine Away from Terminal Ends with More Spacers |
| RP_6_R | 911.5439 | +3 | Effect of Placement of Arginine at the Terminal Ends with More Spacers |
| PPPP | 405.2369 | +1 | Negative Control |
| RG_2_R | 443.2711 | +3 | Effect of Peptide Size |
| RG_4_R | 557.3139 | +3 | Effect of Peptide Size |
| RG_8_R | 785.3995 | +3 | Effect of Peptide Size |
| RG_12_R | 1013.4851 | +3 | Effect of Peptide Size |
| RG_16_R | 1241.5707 | +3 | Effect of Peptide Size |
| RP_2_R | 523.3335 | +3 | Effect of Peptide Size with Bulkier Spacer |
| RP_4_R | 717.4387 | +3 | Effect of Peptide Size with Bulkier Spacer |
| RP_8_R | 1105.6491 | +3 | Effect of Peptide Size with Bulkier Spacer |
| RP_12_R | 1493.8595 | +3 | Effect of Peptide Size with Bulkier Spacer |
| CC | 223.0447 | +1 | Effect of Peptide Size with Cysteine Ends |
| CP_2_C | 417.1499 | +1 | Effect of Peptide Size with Cysteine Ends |
| CP_4_C | 611.2551 | +1 | Effect of Peptide Size with Cysteine Ends |
| CP_8_C | 999.4655 | +1 | Effect of Peptide Size with Cysteine Ends |
| CP_12_C | 1387.6759 | +1 | Effect of Peptide Size with Cysteine Ends |
| (D) NH_2_-R-COOH | 174.1115 | +1 | Effect of D-Conformation |
| (D) RR | 329.2283 | +3 | Effect of D-Conformation |
| HH | 291.1441 | +1 | Effect of pH |
| KK | 273.2159 | +3 | Effect of pH |
| YY | 343.1527 | +1 | Effect of pH |
| YYYY | 669.2789 | +1 | Effect of pH |

III. ESI-MS/MALDI-TOF Mass Spectra of Peptides

**
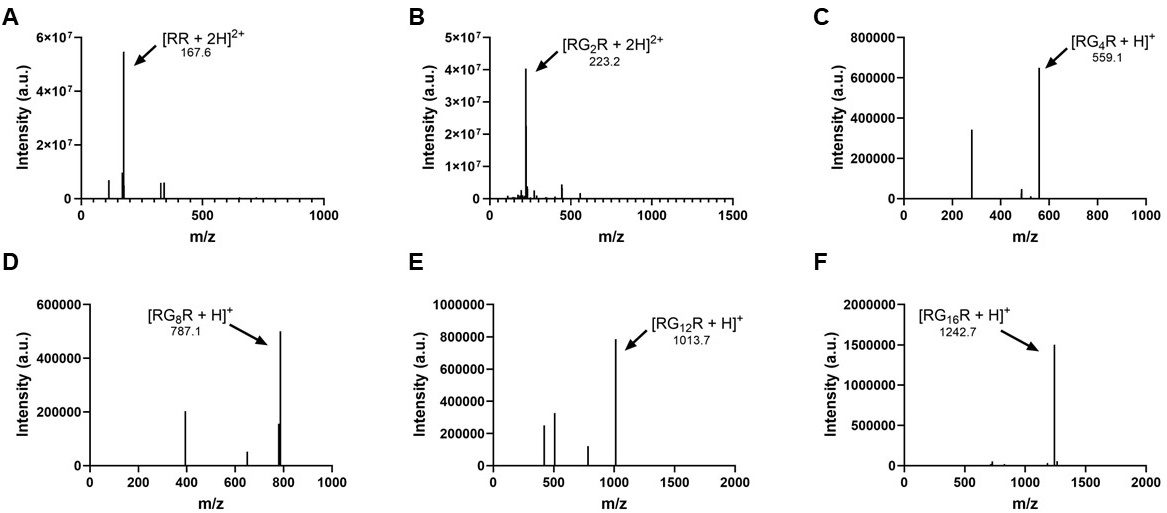
**

**Figure S1.** ESI-MS/MALDI-TOF Mass Spectra of (**A**) RR, (**B**) RG_2_R, (**C**) RG_4_R, (**D**) RG_8_R, (**E**) RG_12_R, and (**F**) RG_16_R.


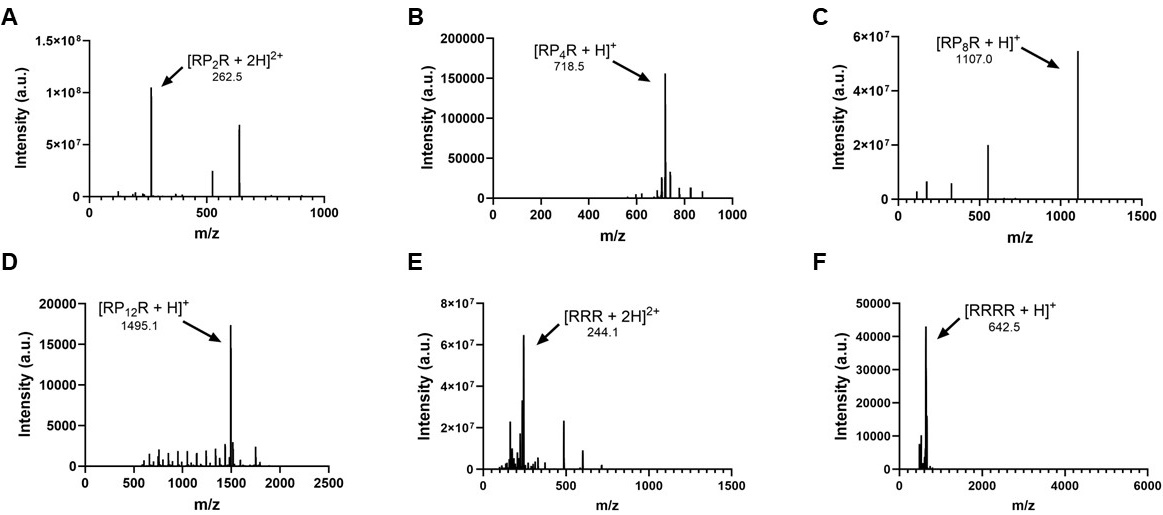


**Figure S2.** ESI-MS/MALDI-TOF Mass Spectra of (**A**) RP_2_R, (**B**) RP_4_R, (**C**) RP_8_R, (**D**) RP_12_R, (**E**) RRR, and (**F**) RRRR.


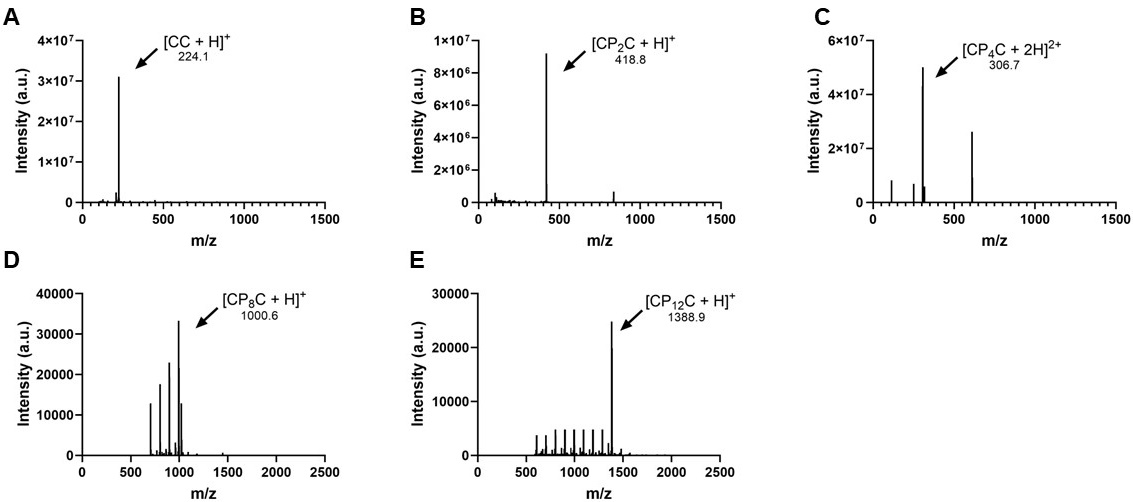


**Figure S3.** ESI-MS/MALDI-TOF Mass Spectra of (**A**) CC, (**B**) CP_2_C, (**C**) CP_4_C, (**D**) CP_8_C, and (**E**) CP_12_C.


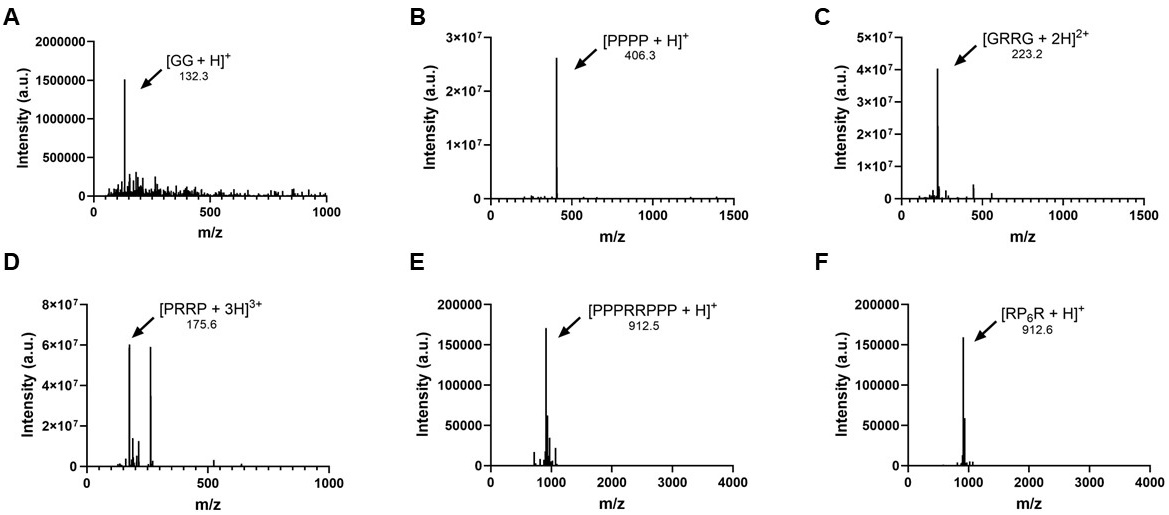


**Figure S4.** ESI-MS/MALDI-TOF Mass Spectra of (**A**) GG, (**B**) PPPP, (**C**) GRRG, (**D**) PRRP, (**E**) PPPRRPPP, and (**F**) RP_6_R.


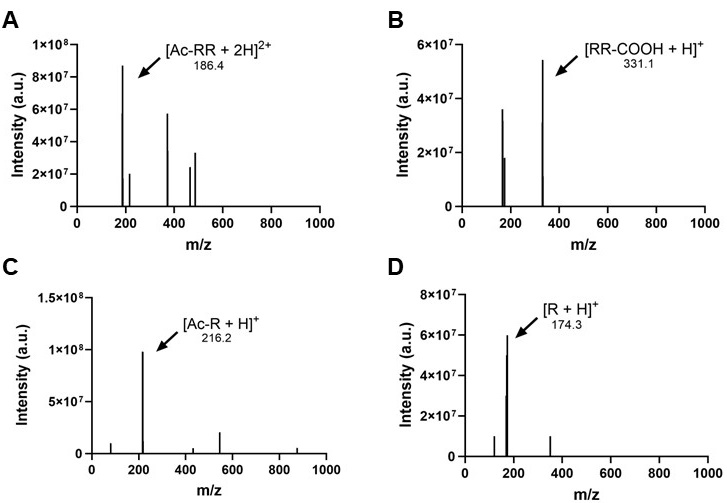


**Figure S5.** ESI-MS/MALDI-TOF Mass Spectra of (**A**) Ac-RR-CONH_2_, (**B**) NH_2_-RR-COOH, (**C**) Ac-R-CONH_2_, and (**D**) R.


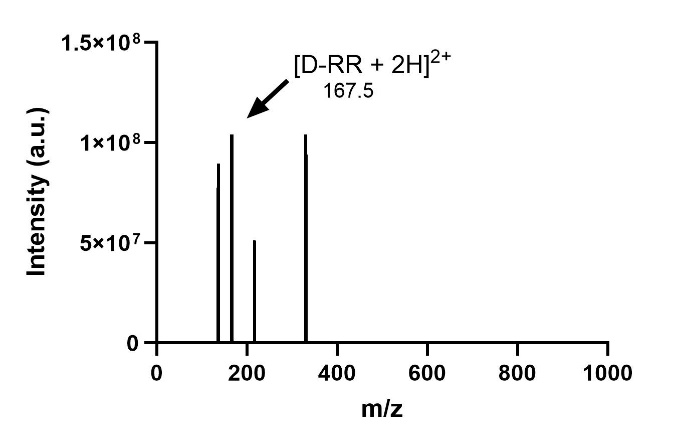


**Figure S6.** ESI-MS/MALDI-TOF Mass Spectra of D-RR.


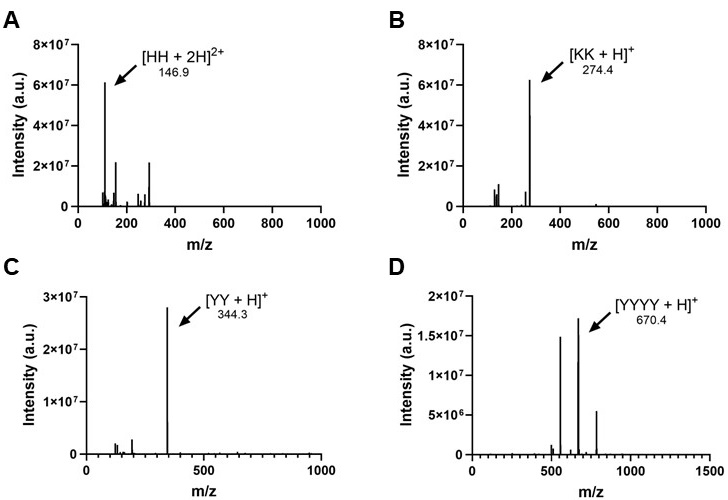


**Figure S7.** ESI-MS/MALDI-TOF Mass Spectra of (**A**) HH, (**B**) KK, (**C**) YY, and (**D**) YYYY.

IV. Size, Absorbance, and Computational Data


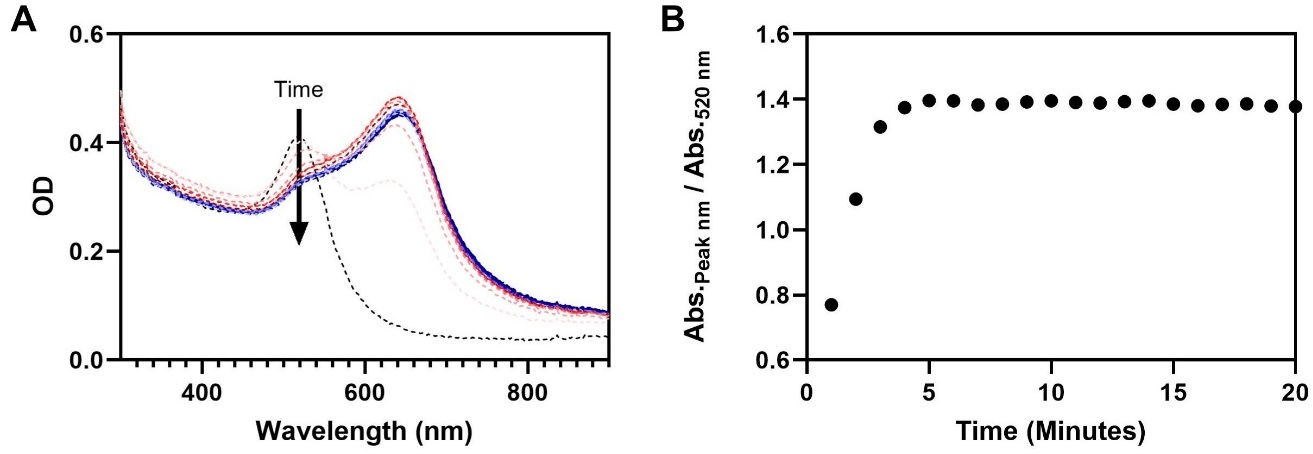


**Figure S8.** (**A**) Kinetic UV-vis spectra indicating the absorbance shift of the AuNPs aggregated by 1 µM RR over 20 minutes. The spectra were measured once every minute. The absorbance spectrum of the AuNPs is shown in black. The spectra change from light red to dark red to dark blue as time progresses. (**B**) Plot showing the increase in ratiometric absorbance over time. The aggregation occurs rapidly within 15 minutes.


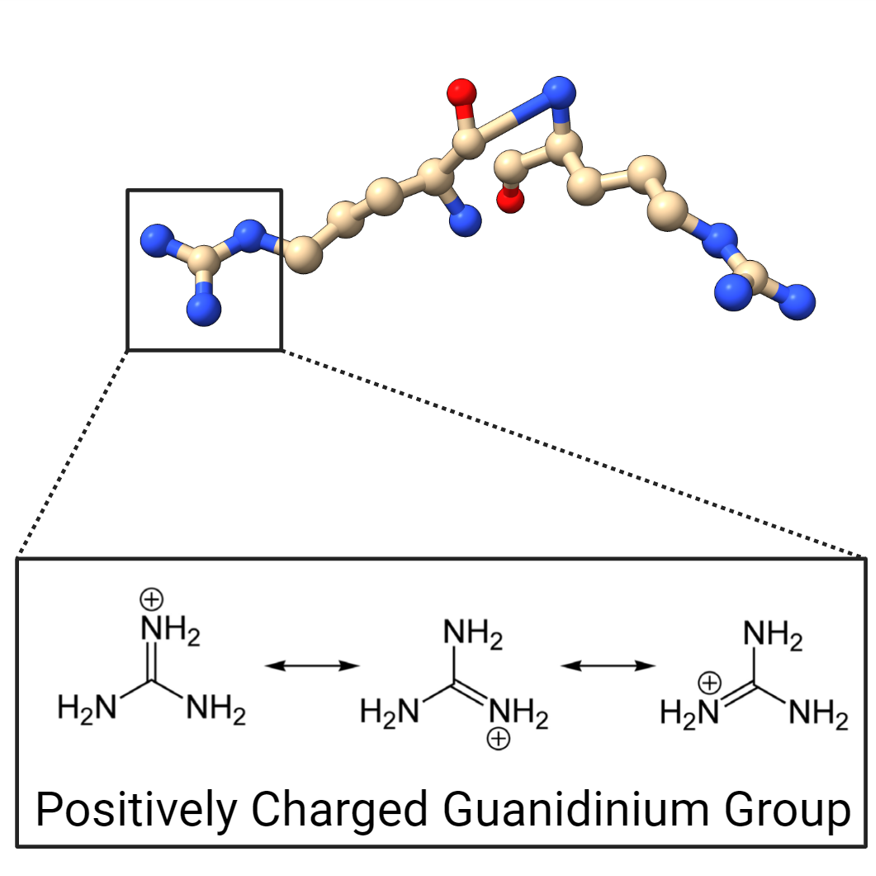


**Figure S9.** Schematic highlighting the positively charged guanidinium group of the cationic RR peptide. Nitrogen atoms are represented in blue, oxygen atoms are represented in red, and carbon atoms are represented in bone.

**
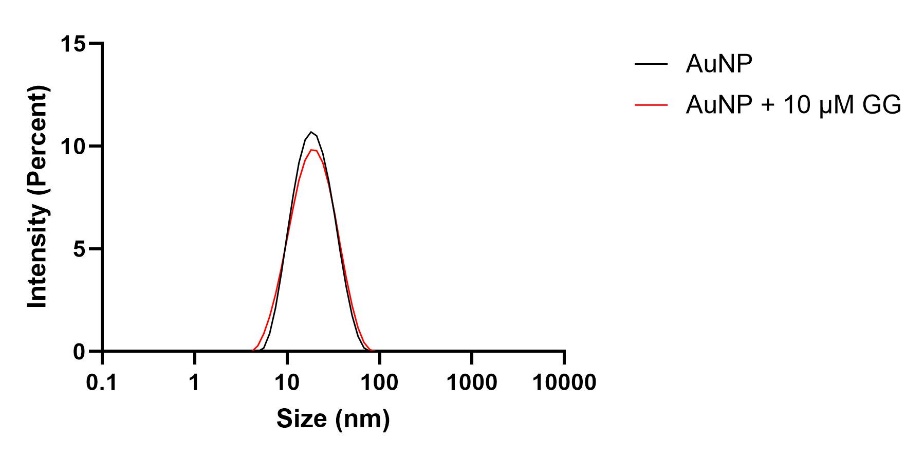
**

**Figure S10.** DLS size spectra comparing the sizes of the AuNPs before and after addition of 10 µM GG.


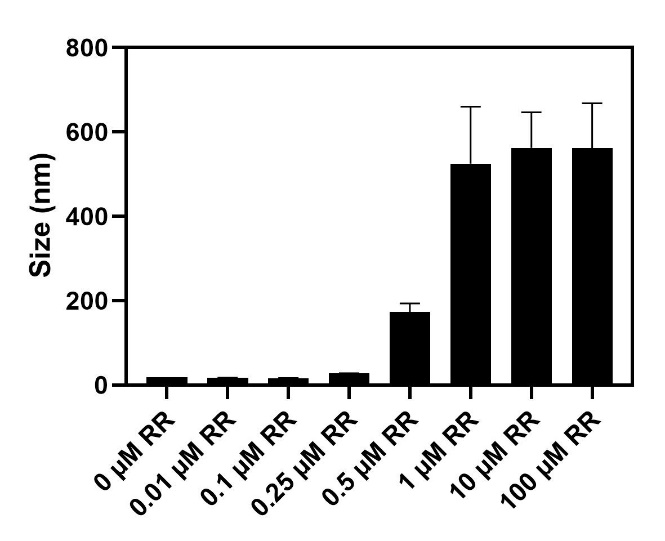


**Figure S11.** Plot showing larger-sized aggregates from DLS measurements as a function of the RR concentration. The error bars represent the standard deviation of three replicates.

**
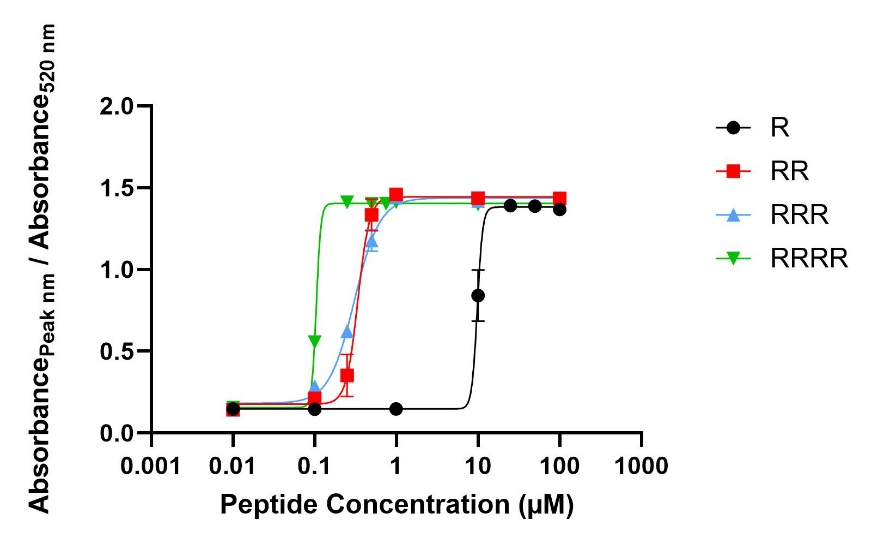
**

**Figure S12.** Dose-response curves comparing the C_50_ values of the R, RR, RRR, and RRRR peptides. The C_50_ value decreases with additional arginine units. The error bars represent the standard deviation of three replicates.


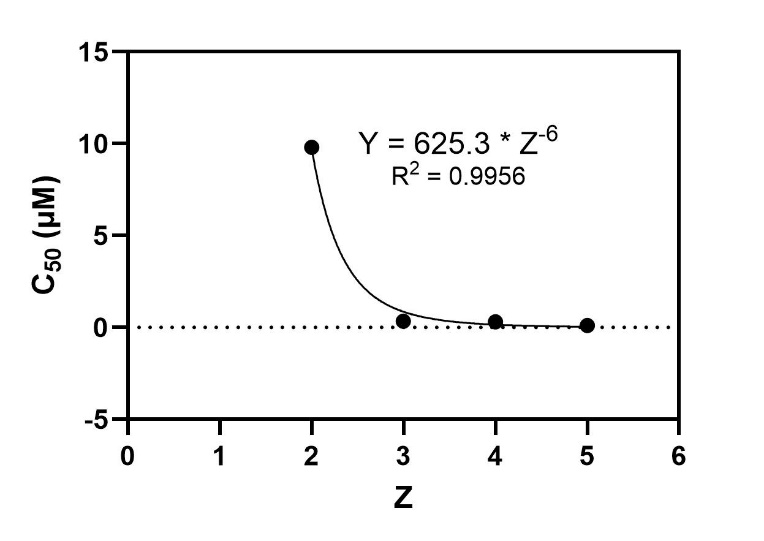


**Figure S13.** Plot showing the C_50_ values of the R, RR, RRR, and RRRR peptides as a function of their net charge (Z). The C_50_ value decreases along the inverse sixth power of the net charge in agreement with the Schulze–Hardy rule. The peptide C_50_ values correspond to the single terminal variant shown in Figure S12.

**Computational Results:**

Calculations were done with CREST and Q-Chem and visualized with VMD.

**
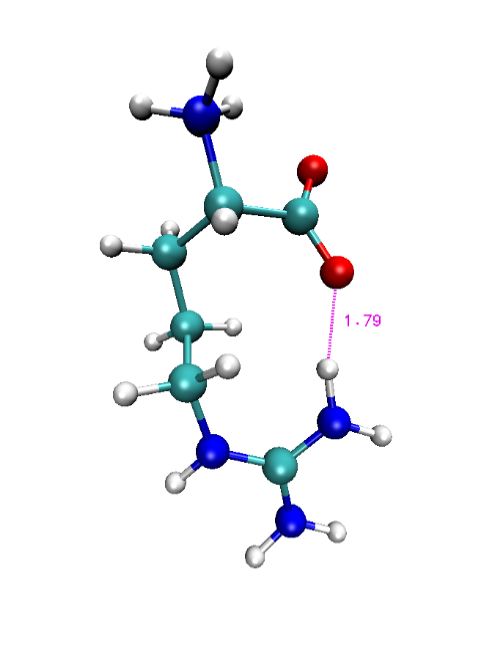

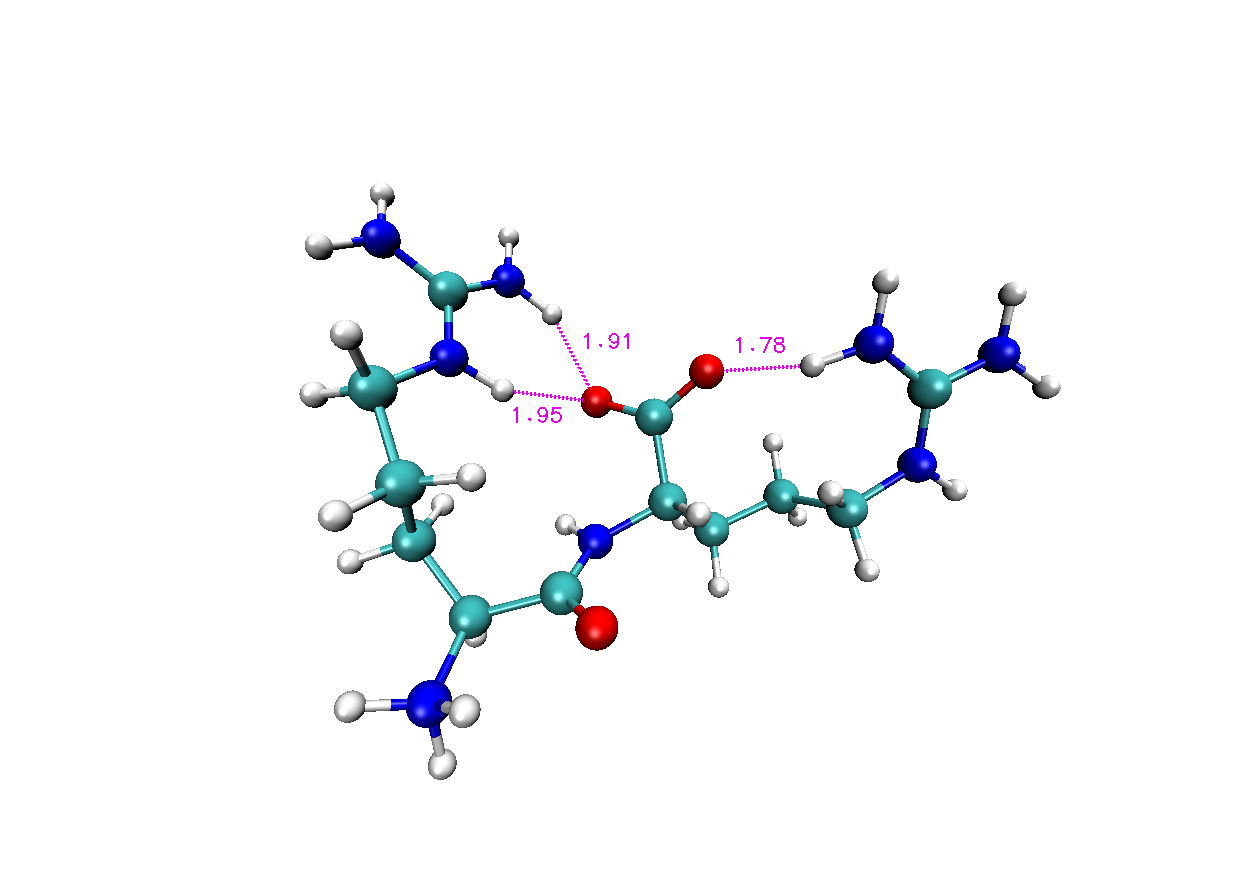
**

**Figure S14.** Structures of NH_2_-R-COOH and NH_2_-RR-COOH showing intramolecular hydrogen bonding. Both oxygen atoms of the carbonyl group in NH_2_-RR-COOH are stabilized by intramolecular hydrogen bonding.


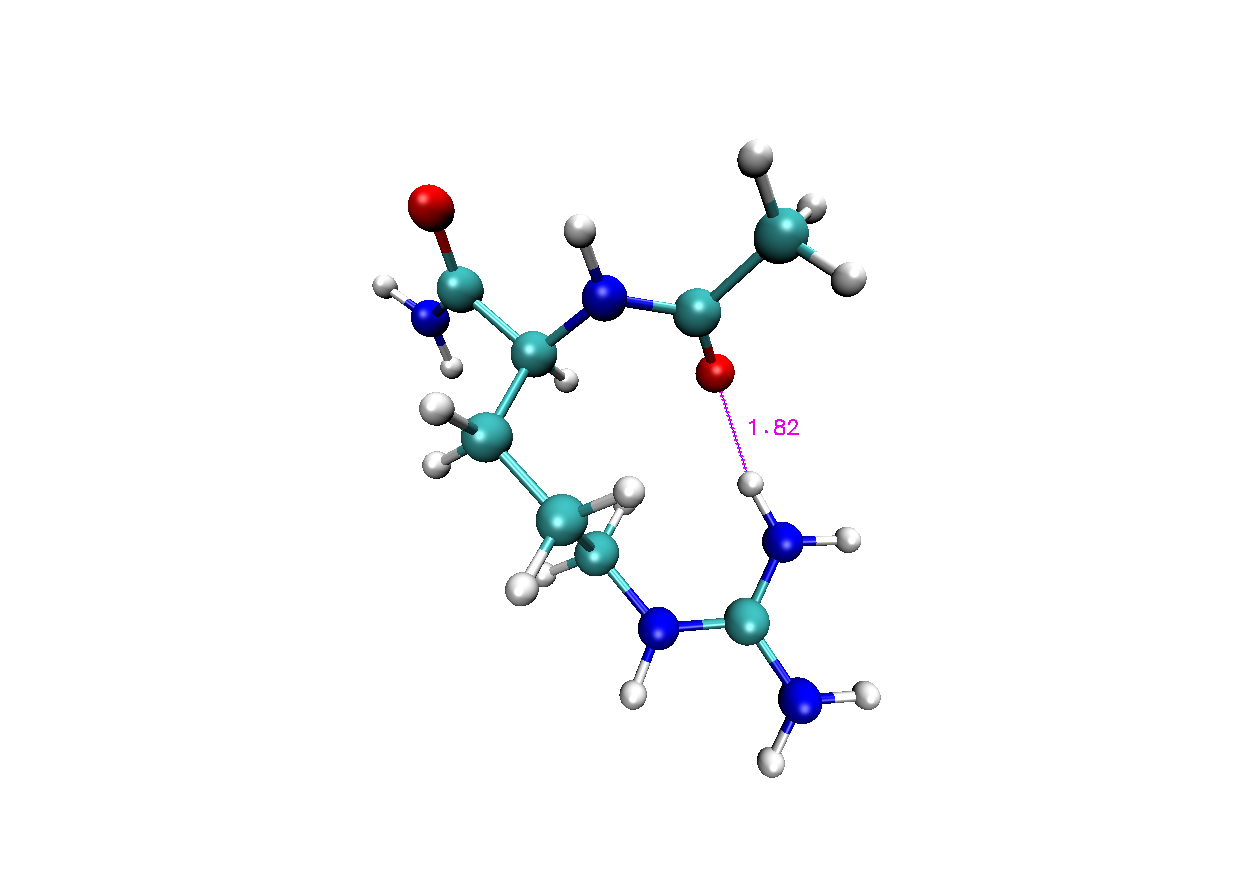

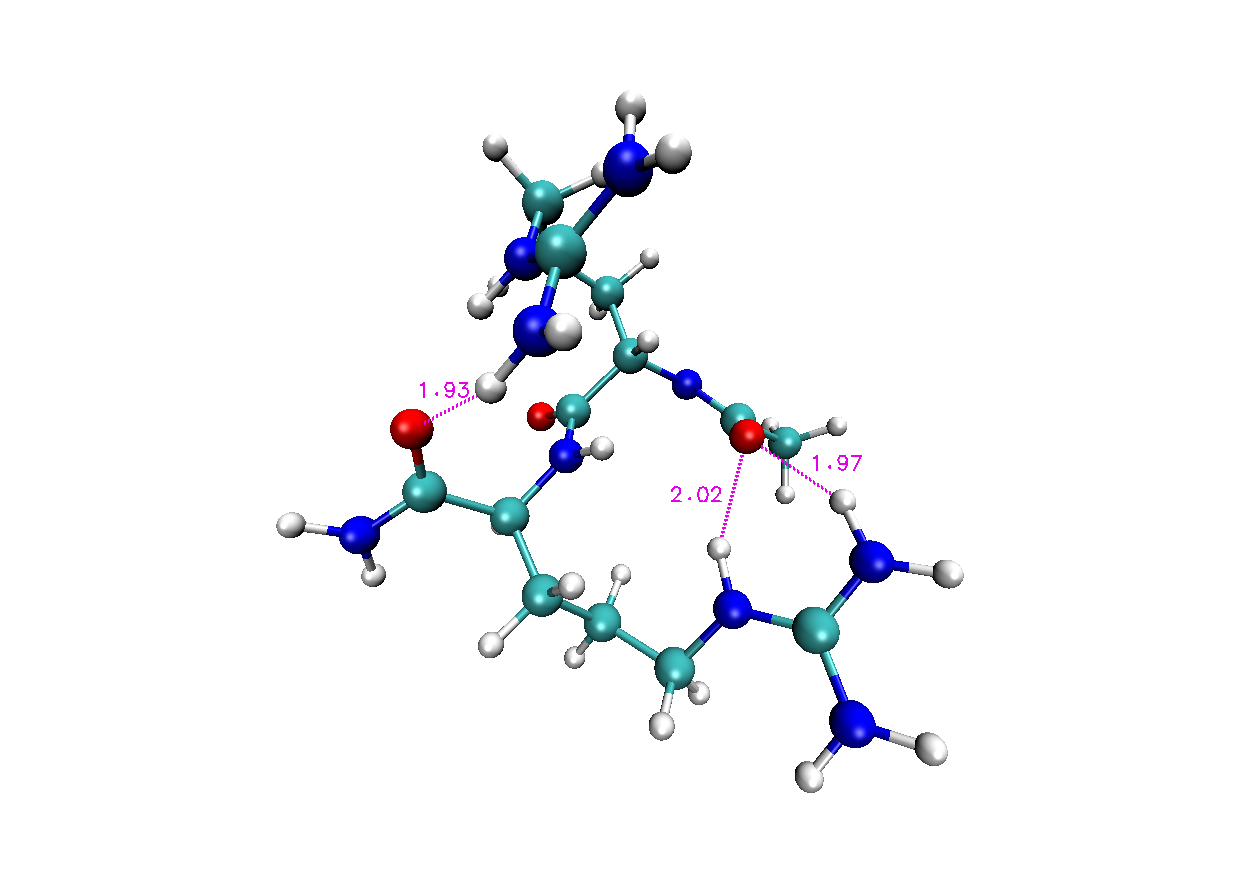


**Figure S15.** Structures of Ac-R-CONH_2_ and Ac-RR-CONH_2_ showing intramolecular hydrogen bonding.


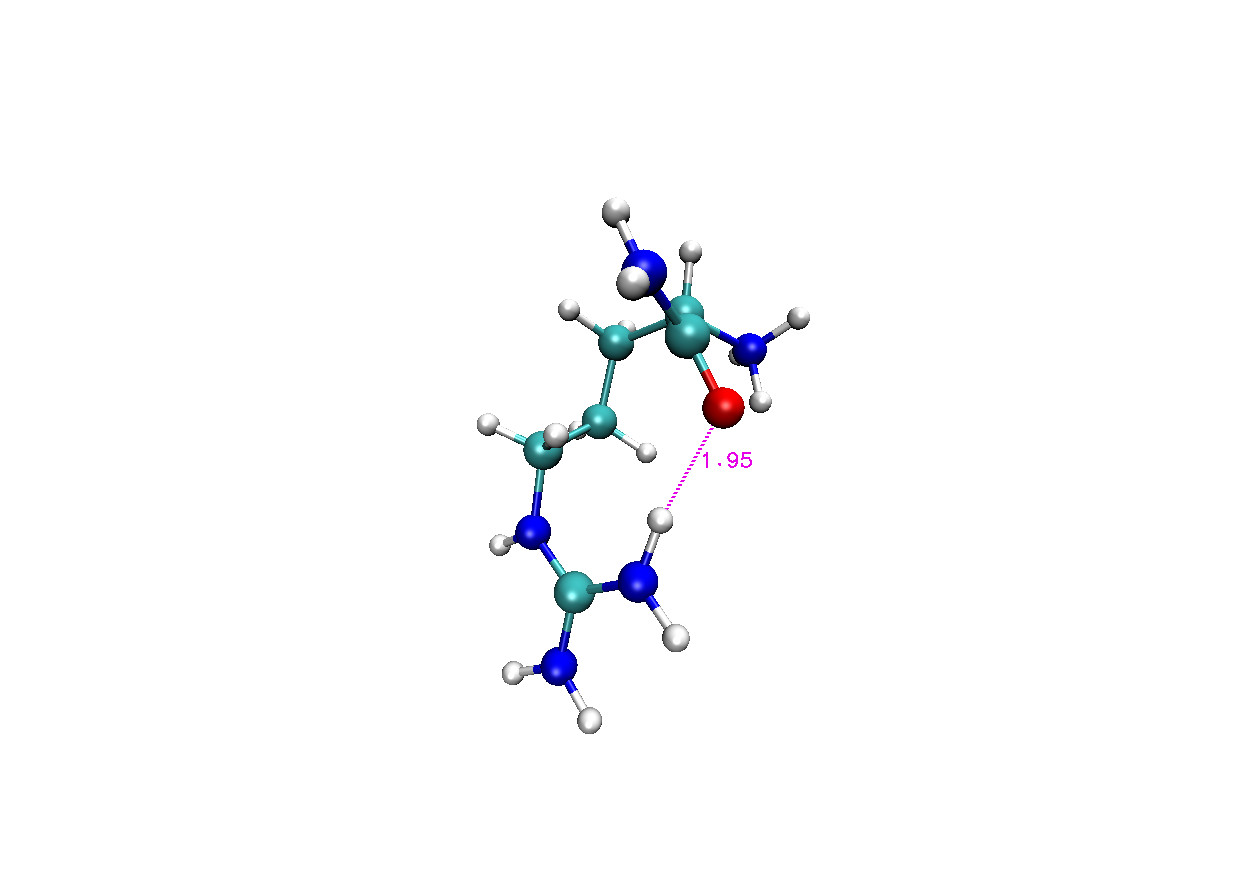

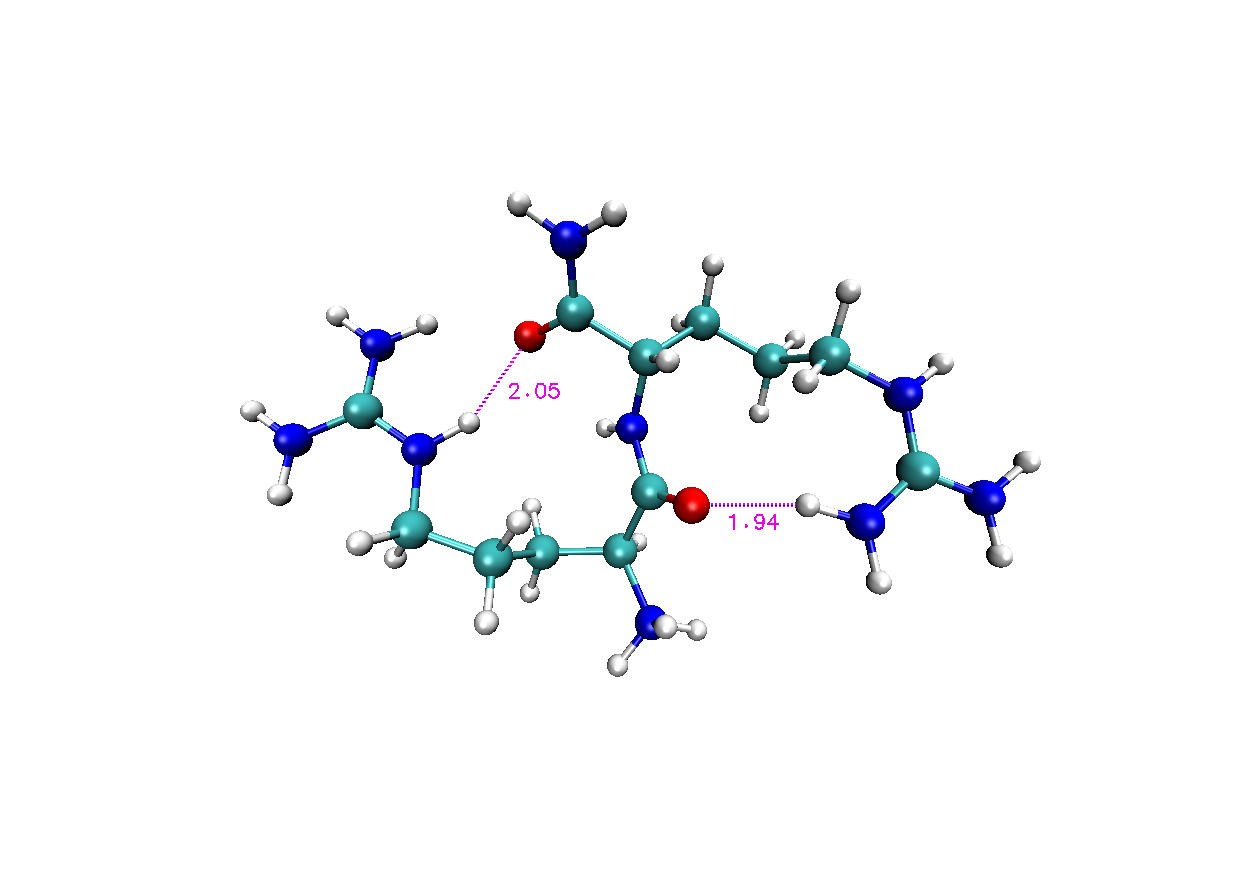


**Figure S16.** Structures of NH_2_-R-CONH_2_ and NH_2_-RR-CONH_2_ showing intramolecular hydrogen bonding.


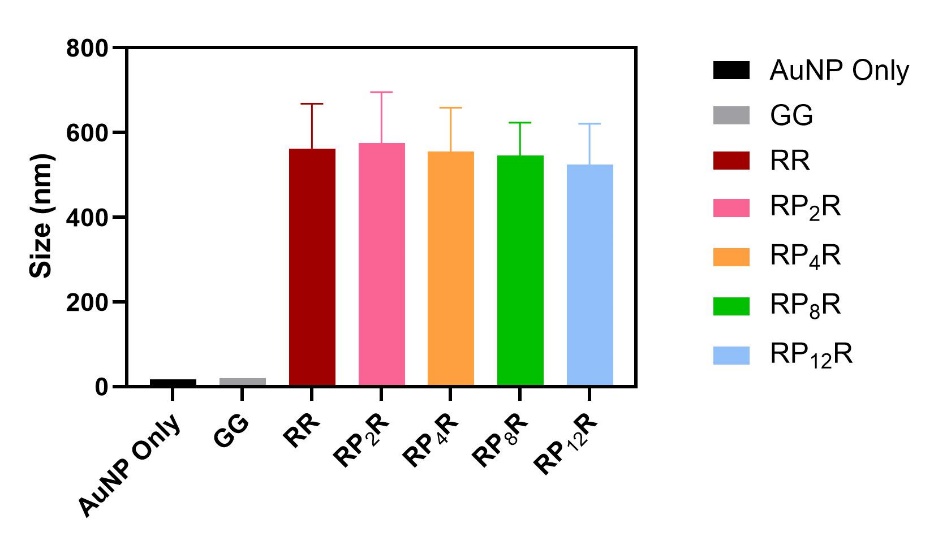


**Figure S17.** Plot comparing the DLS size measurements of the AuNPs after the addition of the GG, RR, RP_2_R, RP_4_R, RP_8_R, and RP_12_R peptides at 100 µM. The error bars represent the standard deviation of three replicates.

**
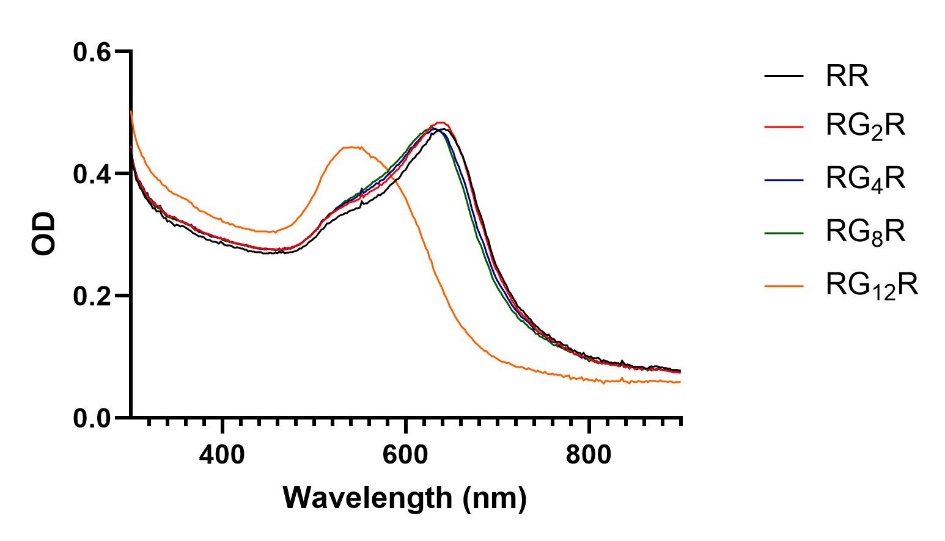
**

**Figure S18.** UV-vis spectra comparing the LSPR peak shift of the RR, RG_2_R, RG_4_R, RG_8_R, and RG_12_R peptides.

V. Peptide Size and Nanoparticle Surface Area Calculations


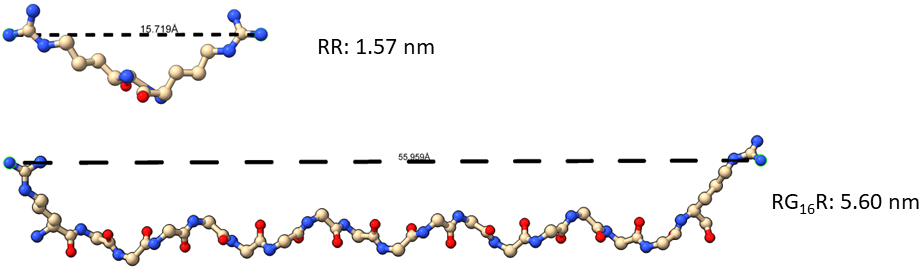


**Figure S19.** Estimated size comparison between the RR and RG_16_R peptides. The peptide structure was predicted using the ESM Metagenomic Atlas and the end-to-end distance of the peptide was measured using ChimeraX.


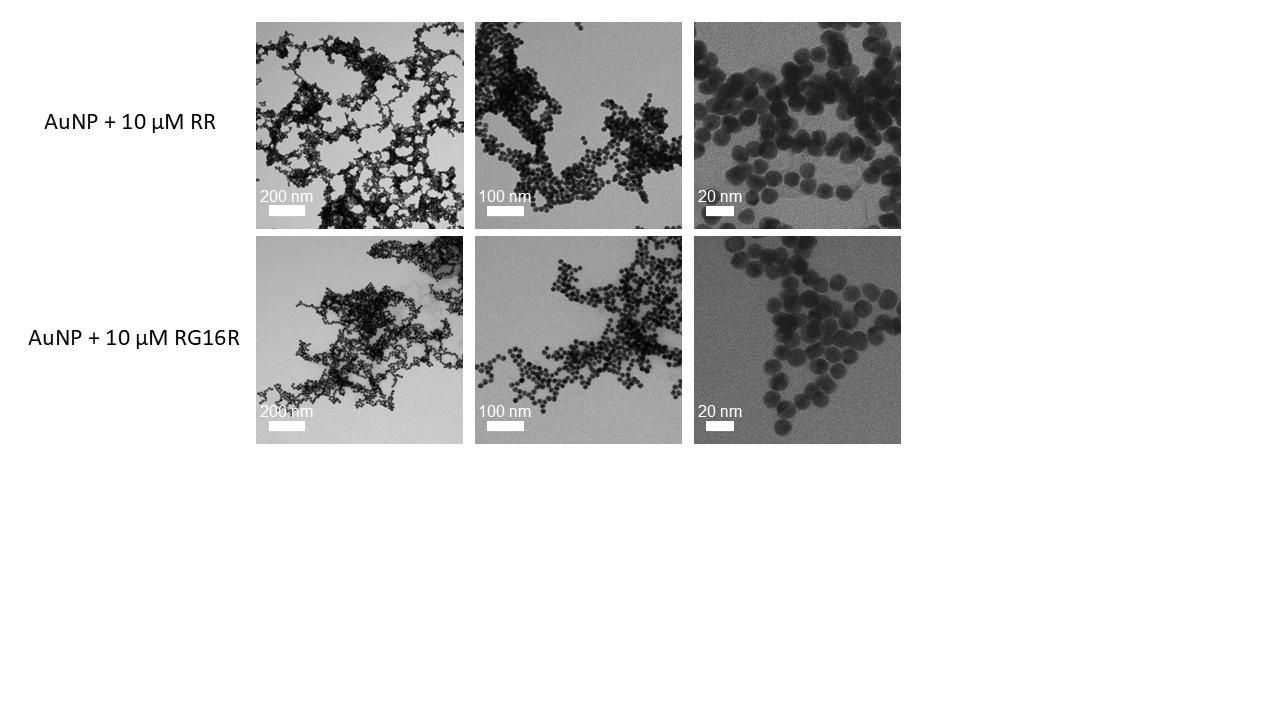


**Figure S20.** TEM images of the AuNPs after aggregation by 10 µM RR and 10 µM RG_16_R peptides. The interparticle distance between the AuNPs appeared to be less than 5 nm for both peptides.

**Fractal Dimension Calculation:**

The fractal dimension was calculated using the getfractaldim() function in MATLAB from the following reference: https://github.com/pranurs/fractal-dimensions. In short, the function provides an automated estimation of the fractal dimension of an image using the box counting method.


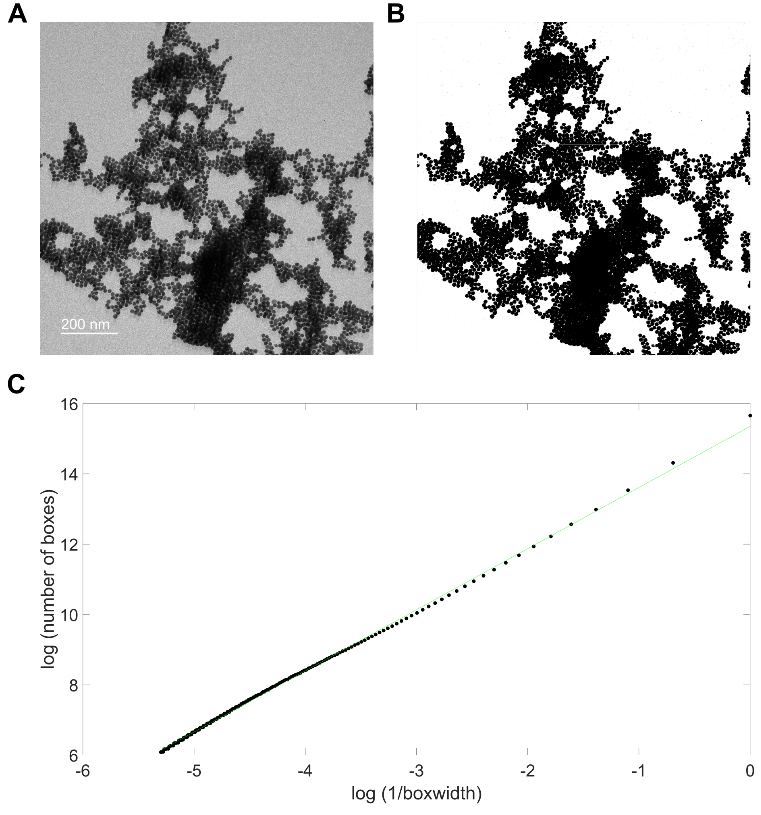


**Figure S21.** (**A**) TEM image of the AuNPs after aggregation by 10 µM RR. (**B**) Contrast-enhanced image produced by the getfractaldim() function to determine the fractal dimension of the cluster. (**C**) Linear regression plot produced by the getfractaldim() function. The slope of the linear regression equation provides the estimate for the fractal dimension (d_f_). The d_f_ was estimated to be 1.7, suggesting diffusion-limited aggregation.


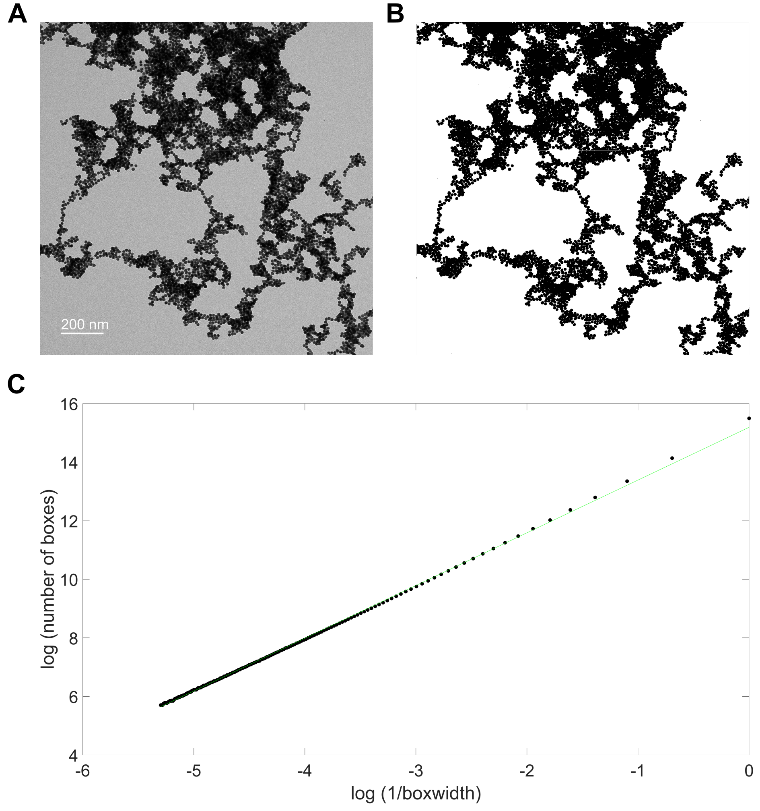


**Figure S22.** (**A**) TEM image of the AuNPs after aggregation by 10 µM RG_16_R. (**B**) Contrast-enhanced image produced by the getfractaldim() function to determine the fractal dimension of the cluster. (**C**) Linear regression plot produced by the getfractaldim() function. The slope of the linear regression equation provides the estimate for the fractal dimension (d_f_). The d_f_ was estimated to be 1.8, suggesting diffusion-limited aggregation.

**
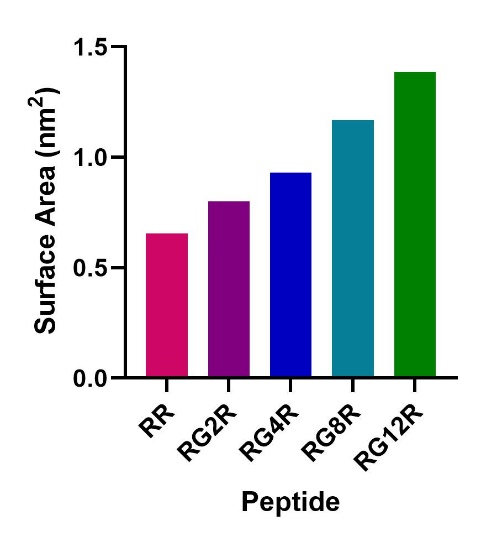
**

**Figure S23.** Estimated surface area coverage of the AuNPs by the RR, RG_2_R, RG_4_R, RG_8_R, and RG_12_R peptides.

**Peptide Surface Area Calculations:**

The theoretical volume of the peptides was estimated from an online peptide calculator from Northwestern University (http://biotools.nu-bic.northwestern.edu/proteincalc.html). The peptide was then approximated as a sphere with that volume to determine its surface area. The surface area was calculated as the cross-sectional area of the sphere. The results are summarized below.

Volume of a Sphere = $V=\frac{4}{3}\pi r^{3}$

Cross-Sectional Area of a Sphere = $A=\pi r^{2}$

| **Peptide** | **Volume (nm^3^)** | **Radius (nm)** | **Surface Area (nm^2^)** |
| --- | --- | --- | --- |
| RR | 0.399 | 0.457 | 0.655 |
| RG_2_R | 0.538 | 0.505 | 0.800 |
| RG_4_R | 0.676 | 0.544 | 0.931 |
| RG_8_R | 0.952 | 0.610 | 1.170 |
| RG_12_R | 1.229 | 0.664 | 1.387 |

**Monolayer Coverage Calculations:**

The theoretical monolayer coverage was calculated by dividing the nanoparticle surface area by the peptide surface area. The nanoparticle surface area was calculated using the surface area formula for a sphere. The calculations are shown below.

Surface Area of a Sphere = 4πr^2^

Surface Area = 4π(9 nm)^2^

Surface Area = 1018 nm^2^

RR: 1018 nm^2^ / 0.655 nm^2^ = 1554 Peptides

RG_2_R: 1018 nm^2^ / 0.800 nm^2^ = 1273 Peptides

RG_4_R: 1018 nm^2^ / 0.931 nm^2^ = 1093 Peptides

RG_8_R: 1018 nm^2^ / 1.170 nm^2^ = 870 Peptides

RG_12_R: 1018 nm^2^ / 1.387 nm^2^ = 734 Peptides

**Predicted Peptide Structure:**

The structures of the peptides were predicted from their amino acid sequence using PEP-FOLD3 (https://bioserv.rpbs.univ-paris-diderot.fr/services/PEP-FOLD3/).


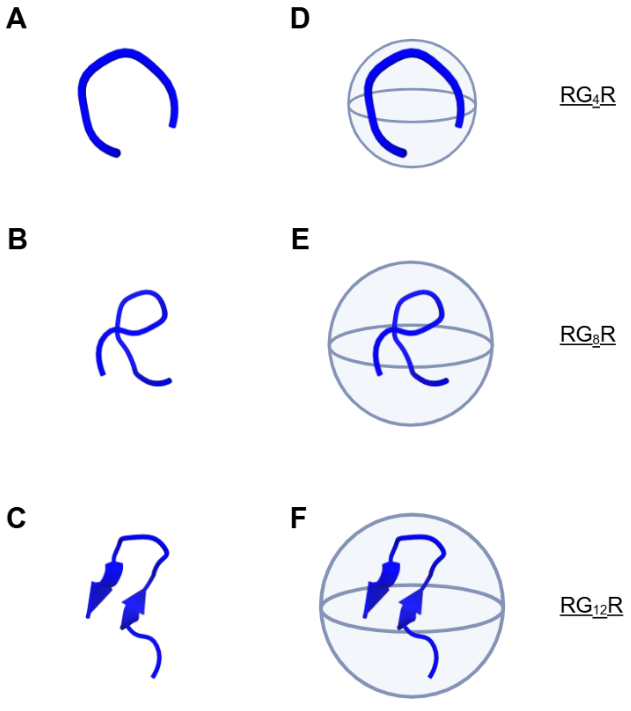


**Figure S24.** Predicted peptide structures using PEP-FOLD3 for the (**A**) RG_4_R, (**B**), RG_8_R, and (**C**) RG_12_R peptides. The structures were overlaid with a sphere (**D-F**), and the cross-sectional area is shown to illustrate the approximate peptide surface area.

VI. Characterization of AuNP Assembly and Dissociation


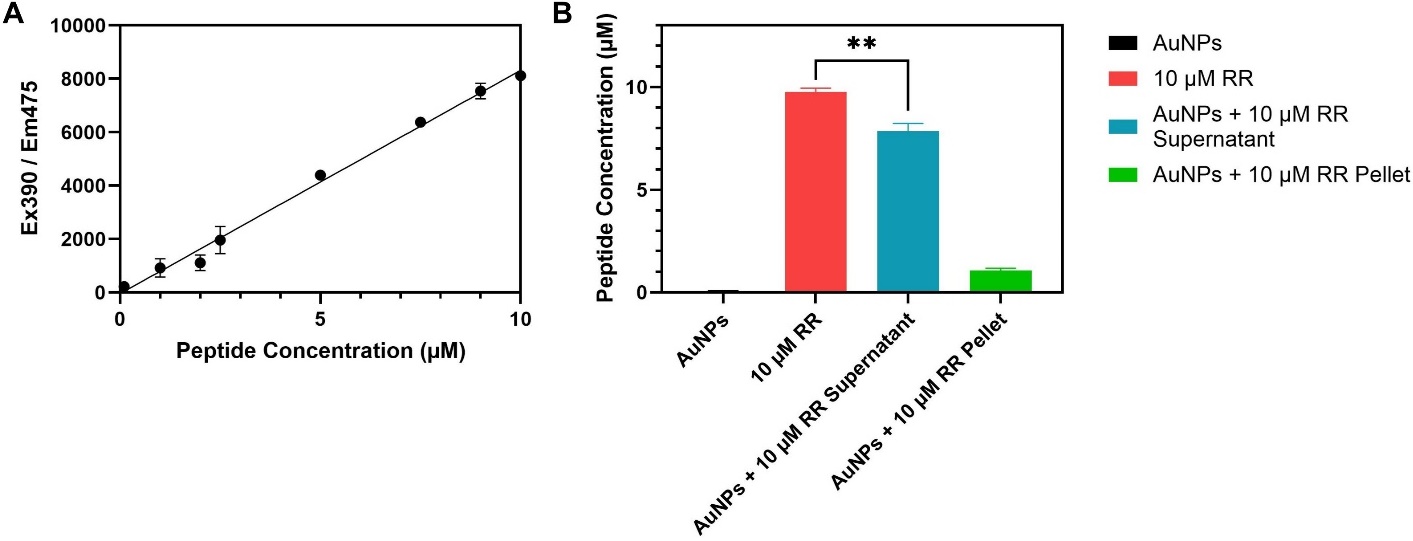


**Figure S25.** (**A**) Standard curve for the quantification of the RR peptide using the Pierce Quantitative Fluorometric Peptide Assay. (**B**) Plot comparing the peptide concentrations measured using the assay. The decrease in peptide concentration in the supernatant suggests that 2.1 µM of the peptide is adsorbed to the surface of the AuNPs. All error bars represent the standard deviation of three replicates. Asterisks indicate statistical significance using Student’s t test (**p < 0.01).


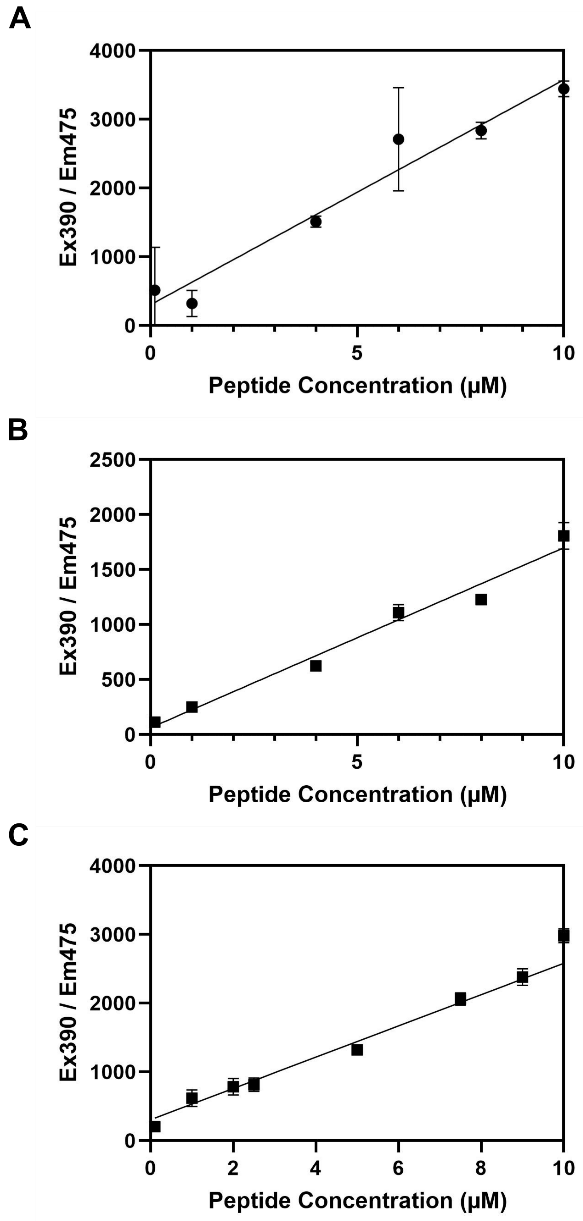


**Figure S26.** Standard curves for the quantification of the (**A**) RG_4_R, (**B**) RG_8_R, and (**C**) RG_12_R peptides using the Pierce Quantitative Fluorometric Peptide Assay. All error bars represent the standard deviation of three replicates.


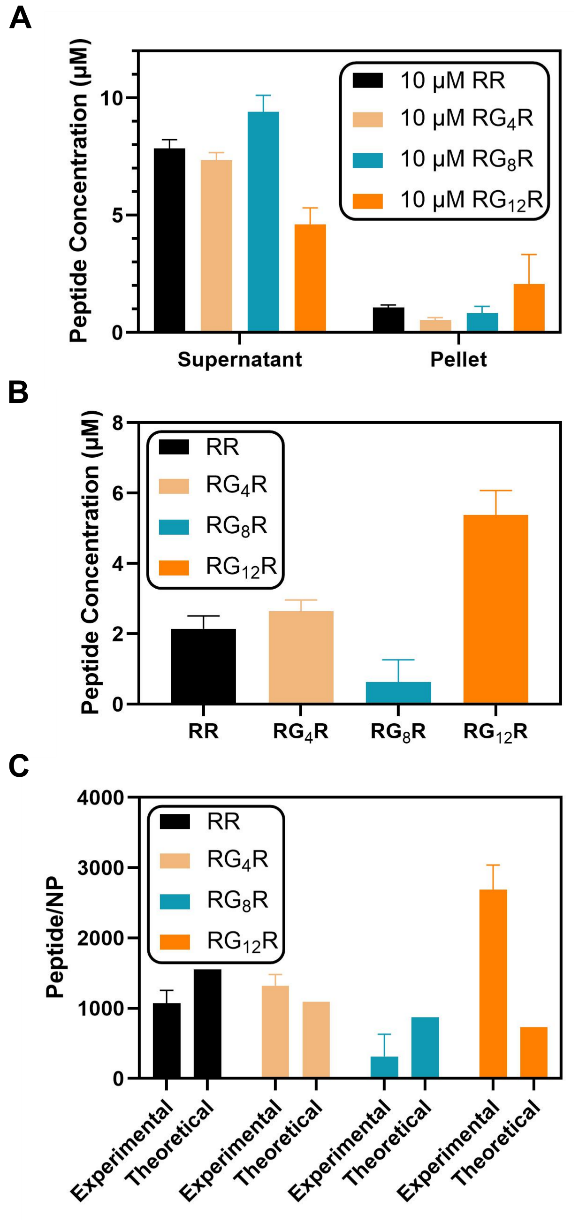


**Figure S27.** (**A**) Plot showing the measured peptide concentrations using the Pierce Quantitative Fluorometric Peptide Assay. The concentration of peptide remaining in the supernatant generally decreases for the peptides with more glycine spacer units. The concentration of peptide in the pellet plus the concentration in the supernatant approximately equals the 10 µM added but generally underestimates the total. This suggests that the peptides in the pellet may be undercounted due to loss of the peptide during removal of the supernatant or unaccounted peptides within the aggregate. (**B**) Plot comparing the peptide concentrations adsorbed onto the AuNPs by subtracting the amount in the supernatant from the 10 µM added. (**C**) Plot comparing the number of peptides per AuNP adsorbed relative to the theoretical monolayer coverage. The experimental peptide densities were all roughly within the same order of magnitude as the theoretical approximation. All error bars represent the standard deviation of three replicates.


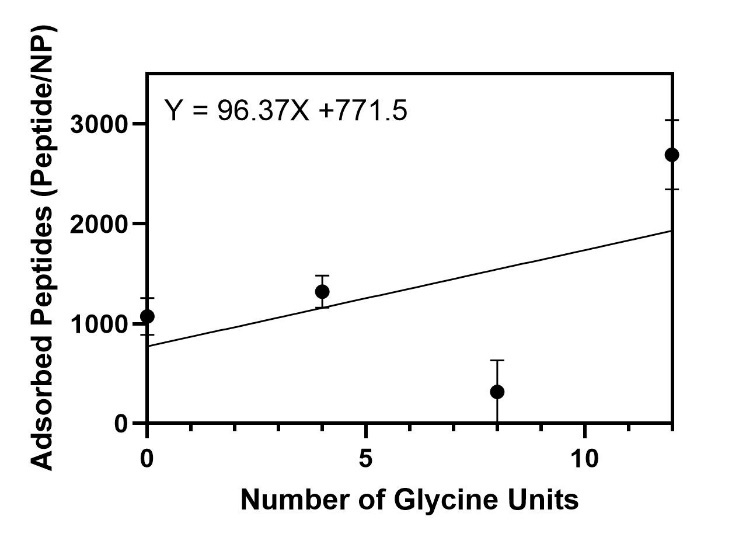


**Figure S28.** Linear regression analysis of the adsorbed peptide density as a function of the number of glycine spacer units in the peptide. The slope indicates that the peptide density adsorbed onto the AuNPs increases by about 100 peptides per glycine spacer unit. The error bars represent the standard deviation of three replicates.


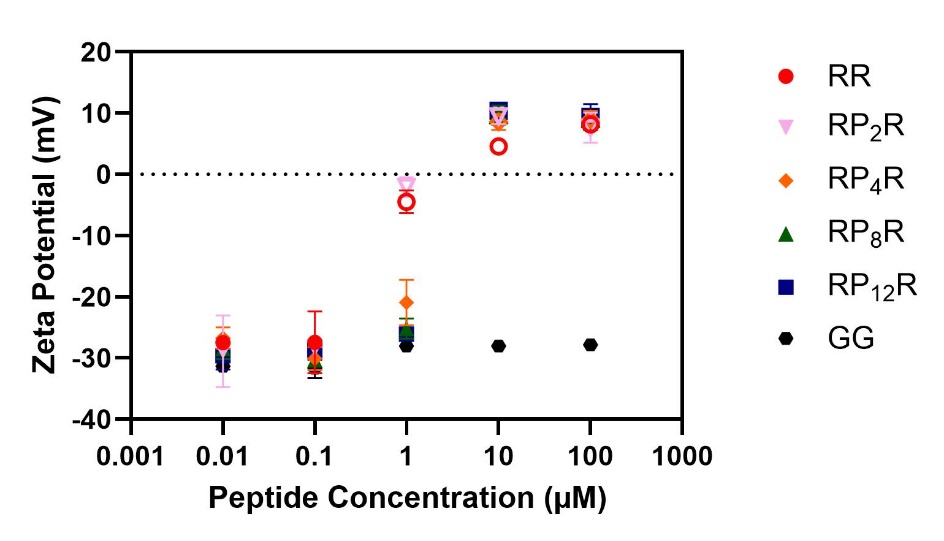


**Figure S29.** Plot illustrating the zeta potential as a function of the peptide concentration for the RR, RP_2_R, RP_4_R, RP_8_R, RP_12_R, and GG peptides. Open symbols indicate aggregated AuNPs, while closed symbols indicate unaggregated AuNPs. The error bars represent the standard deviation of three replicates.


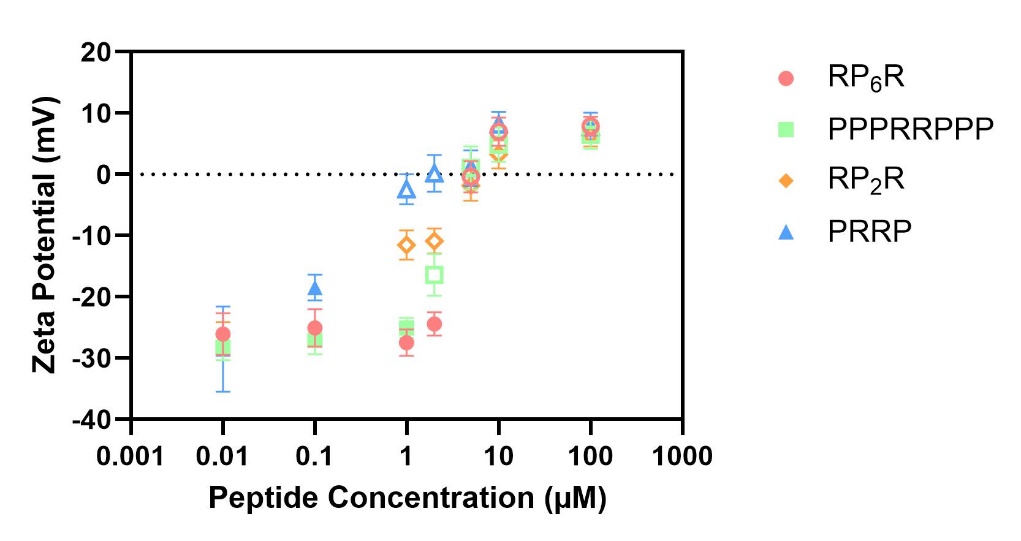


**Figure S30.** Plot illustrating the zeta potential as a function of the peptide concentration for the RP6R, PPPRRPPP, RP_2_R, and PRRP peptides. The peptides with central grafting points (PPPRRPPP and PRRP) had a stronger increase in the zeta potential. Open symbols indicate aggregated AuNPs, while closed symbols indicate unaggregated AuNPs. The error bars represent the standard deviation of three replicates.


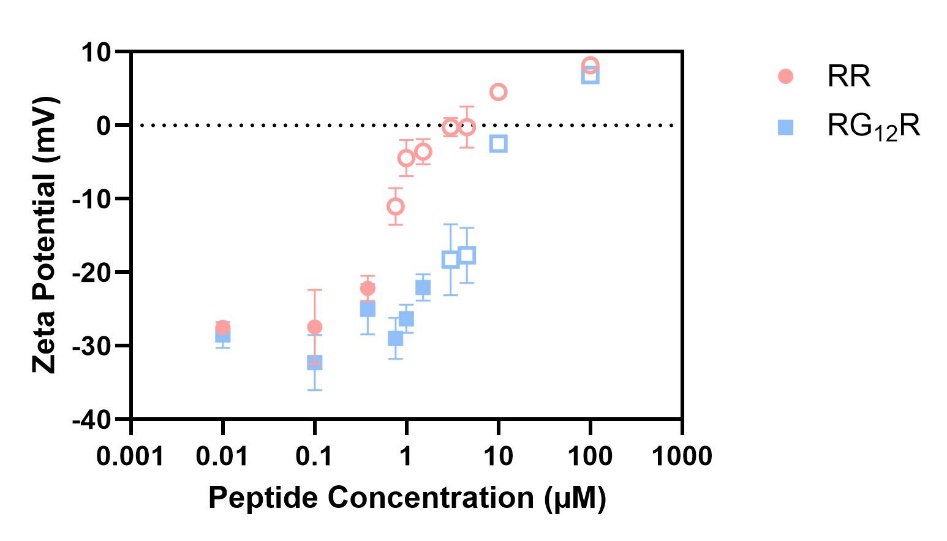


**Figure S31.** Plot illustrating the zeta potential as a function of the peptide concentration for the RR and RG_12_R peptides in finer detail. RR was more effective in modifying the surface charge of the AuNPs, requiring less peptide per nanoparticle for a given potential change. Open symbols indicate aggregated AuNPs, while closed symbols indicate unaggregated AuNPs. The error bars represent the standard deviation of three replicates.


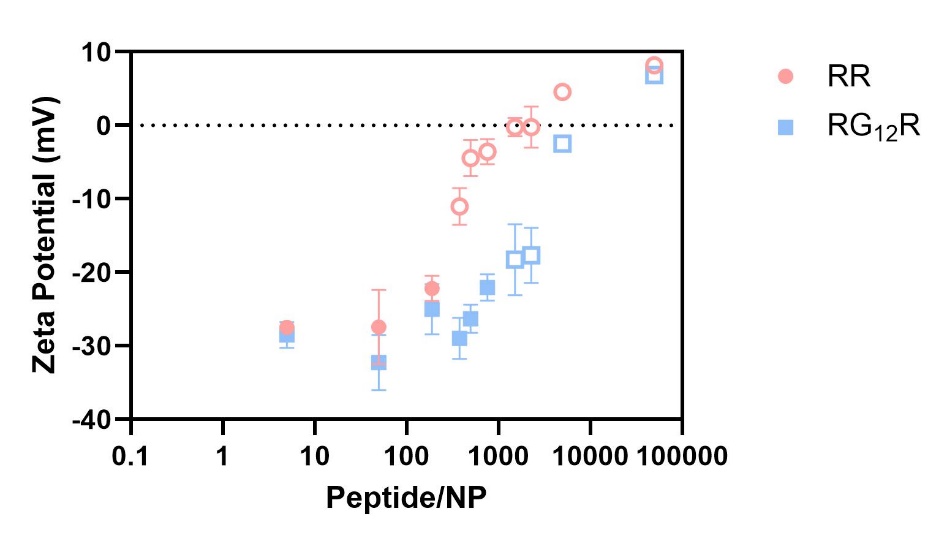


**Figure S32.** Plot illustrating the zeta potential as a function of the peptide concentration for the RR and RG_12_R peptides in units of Peptide/NP. RR was more effective in modifying the surface charge of the AuNPs, requiring less peptide per nanoparticle for a given potential change. Open symbols indicate aggregated AuNPs, while closed symbols indicate unaggregated AuNPs. The error bars represent the standard deviation of three replicates.

**
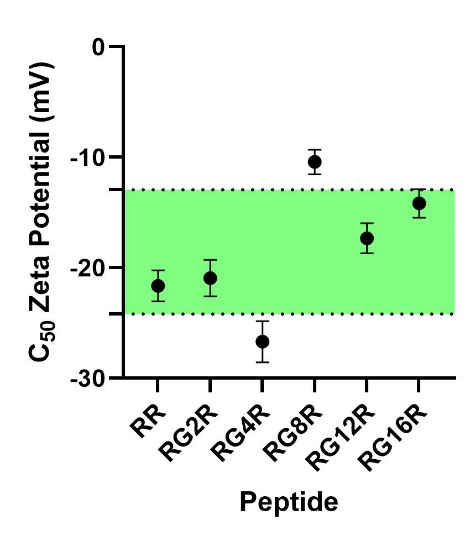
**

**Figure S33.** Zeta potential measurements of the AuNPs after aggregation by the RR, RG_2_R, RG_4_R, RG_8_R, and RG_12_R peptides at the C_50_ concentration. Error bars represent the standard deviation of three replicates.


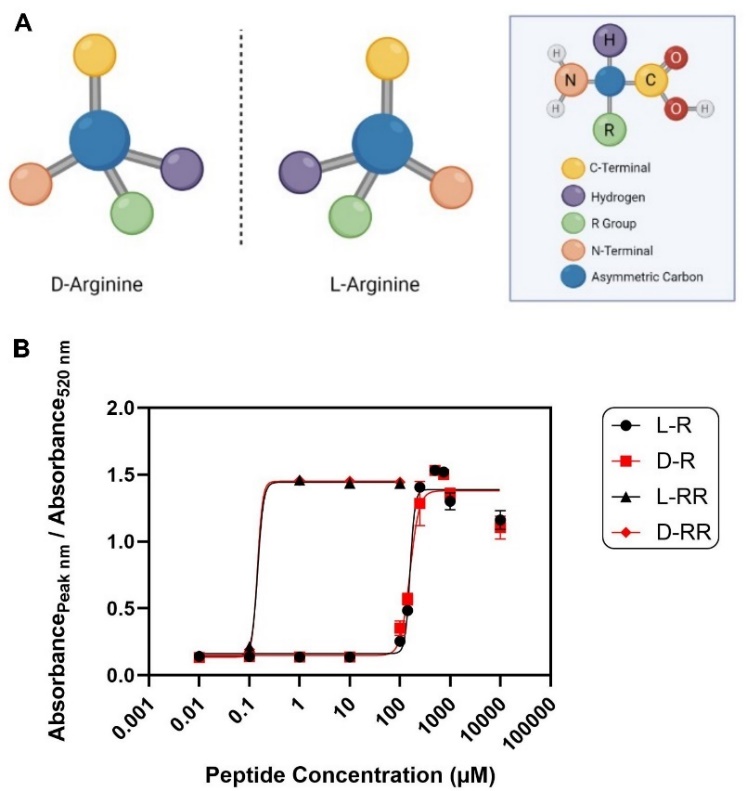


Figure S34. Effect of the peptide stereochemistry. (A) Schematic illustrating the differences in stereochemistry of D-arginine and L-arginine. (B) Dose-response curves showing that the stereochemistry of the arginine group does not have a substantial effect on the quantity of peptide required to induce aggregation of the AuNPs. Error bars represent the standard deviation of three replicates.


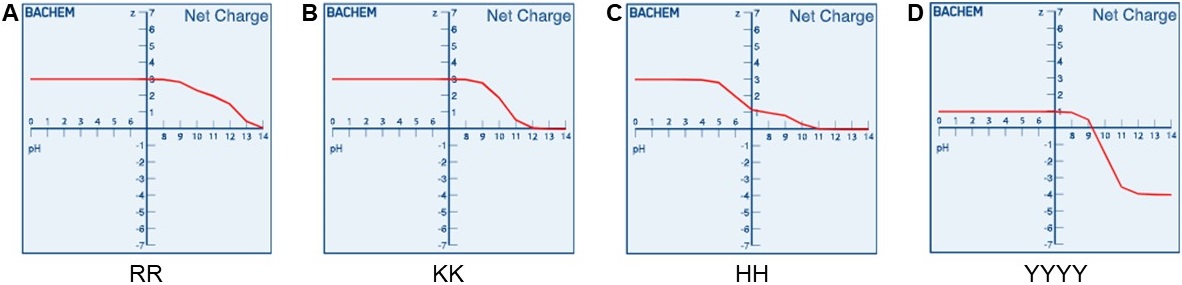


Figure S35. Net charge of the (A) RR, (B) KK, (C) HH, and (D) YYYY peptides as a function of pH. The data was acquired from BACHEM (https://www.bachem.com/knowledge-center/peptide-calculator/).


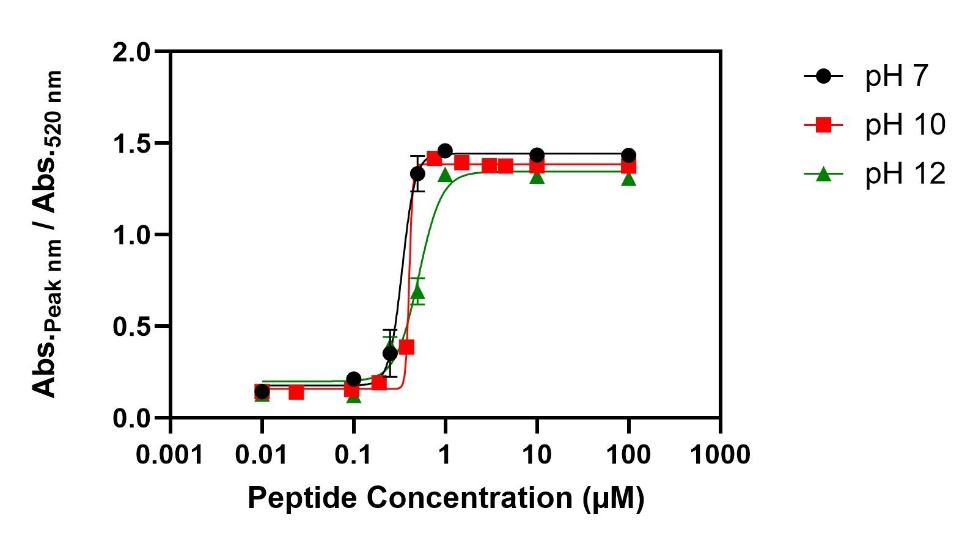


Figure S36. Dose-response curves for the RR peptide at pH 7, 10, and 12. The larger C_50_ increase from pH 10 to 12 suggests that the deprotonation of the arginine residues has a greater effect than the deprotonation of the N-terminal amines. The error bars represent the standard deviation of three replicates.


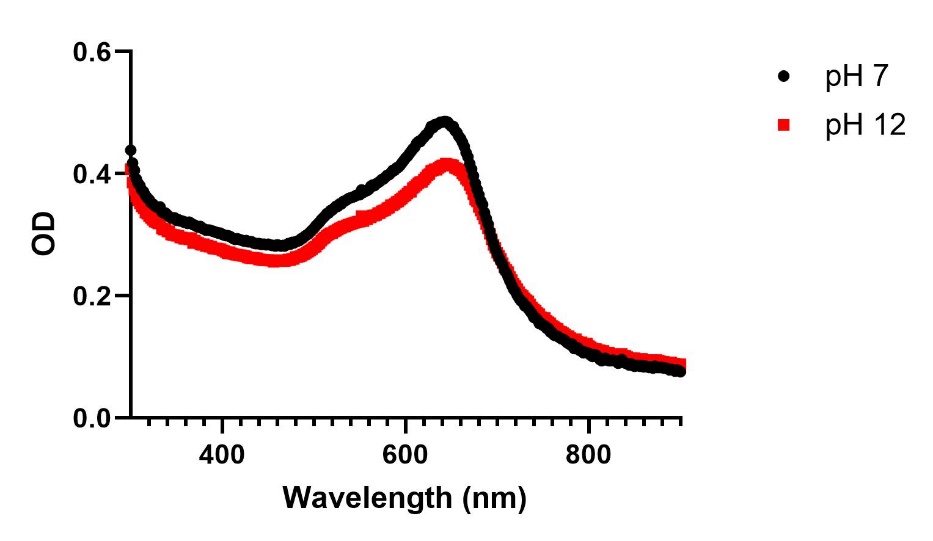


Figure S37. UV-vis spectra indicating the lack of the dissociation of the AuNPs at pH 12 after aggregation by the KK peptide at pH 7.


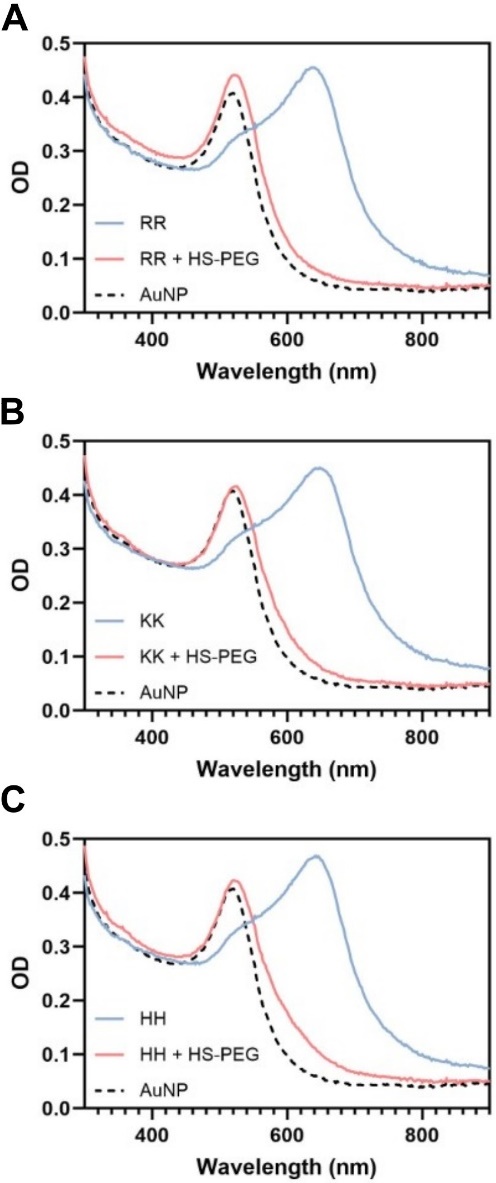


**Figure S38.** UV-vis spectra indicating reversible aggregation of the AuNPs upon addition of 10 µM HS-PEG for the (**A**) RR-, (**B**) KK-, and (**C**) HH-aggregated AuNPs.

**
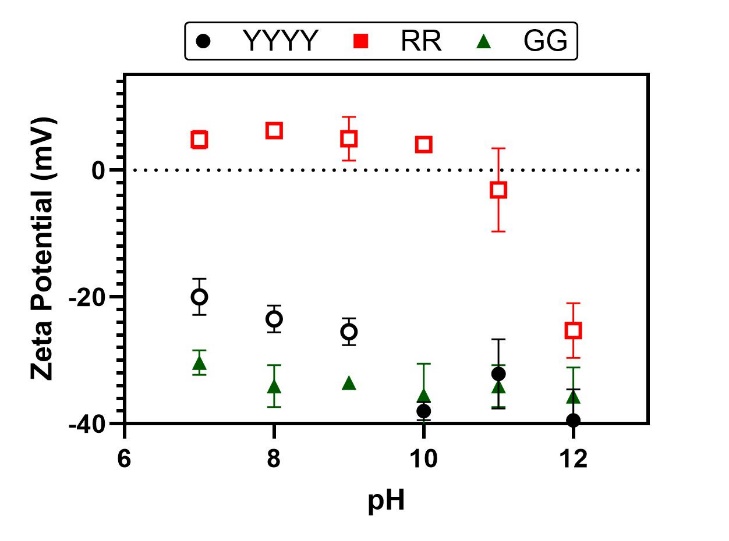
**

**Figure S39.** Zeta potential measurements of the AuNPs at increasing pH values. Open symbols indicate aggregated AuNPs, while closed symbols indicate unaggregated AuNPs. The error bars represent the standard deviation of three replicates.

**
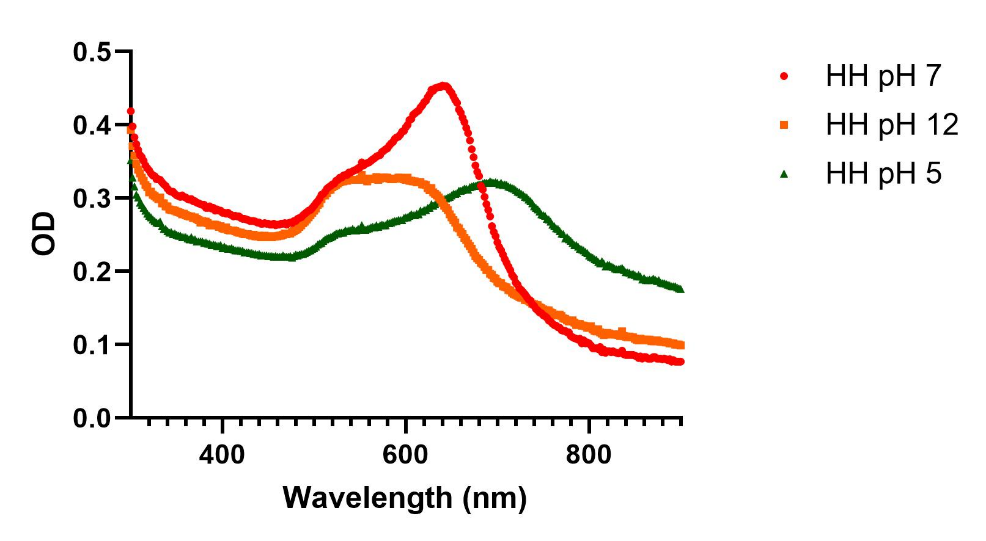
**

**Figure S40.** UV-vis spectra indicating the reversible dissociation and aggregation of the AuNPs by changing the pH to 12 and 5 after aggregation by the HH peptide at pH 7.

**
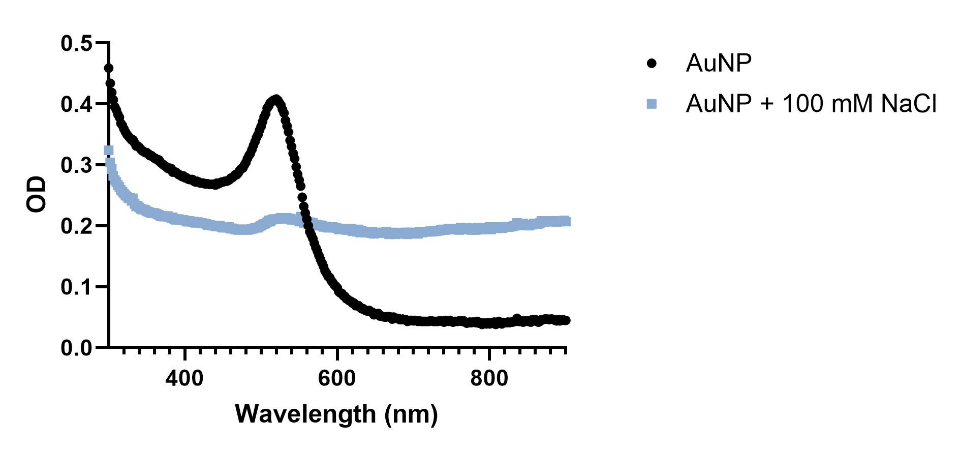
**

**Figure S41.** UV-vis spectra indicating the aggregation of the AuNPs without addition of any peptide at 100 mM NaCl.

**
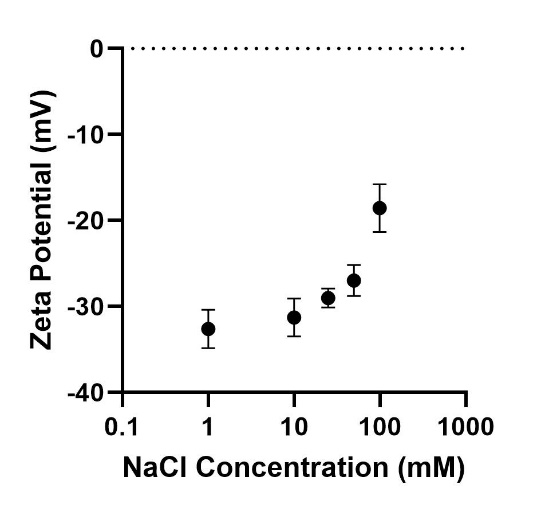
**

**Figure S42.** Plot illustrating the zeta potential of the AuNPs as a function of the NaCl concentration added. The AuNPs were no longer colloidally stable at 100 mM NaCl due to diminished electrostatic repulsion. The error bars represent the standard deviation of three replicates.

**
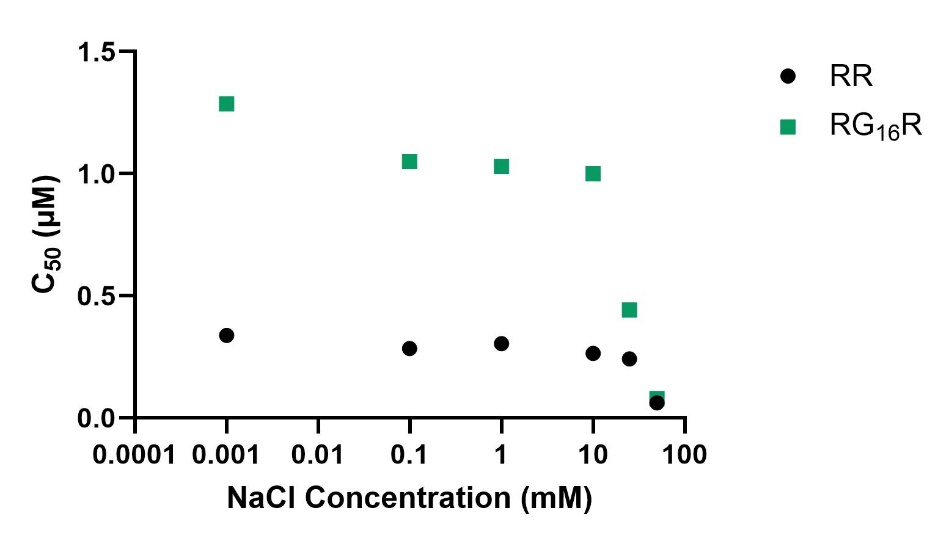
**

**Figure S43.** Plot comparing the C_50_ values for the RR and RG_16_R peptides as a function of the NaCl concentration.

**
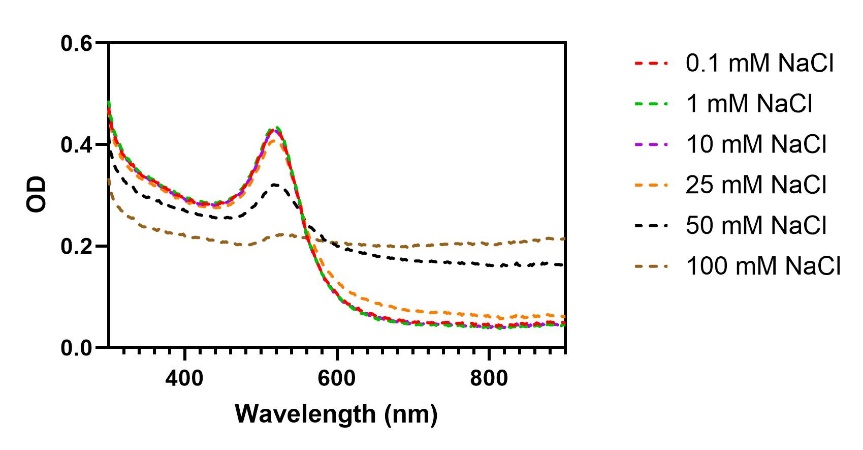
**

**Figure S44.** UV-vis spectra indicating the relative stability of the AuNPs at NaCl concentrations below 50 mM.


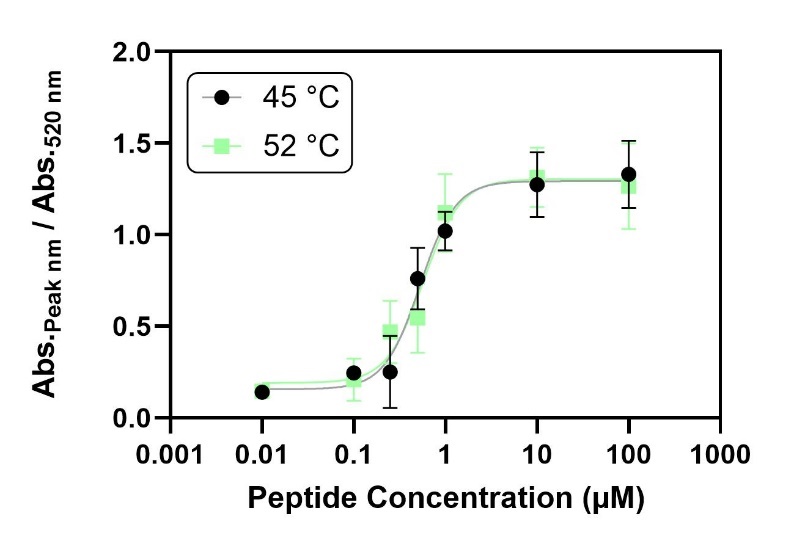


**Figure S45.** Dose-response curves for the RR peptide at temperatures of 45 °C and 52 °C. The error bars represent the standard deviation of three replicates.

**Temperature Sensitivity Calculation:**

The C_50_ temperature sensitivity calculation was adapted from the following reference:

Gustafson, T. P.; Cao, Q.; Wang, S. T.; Berezin, M. Y. Design of Irreversible Optical Nanothermometers for Thermal Ablations. *Chem*. *Commun*. **2013**, *49* (7), 680–682. https://doi.org/10.1039/C2CC37271A.

The following equations were used in the calculation:

- $S\left( T \right)=\frac{\Delta F\left( T \right)}{dT}\times100\%$
- $\Delta F=\frac{F_{T}-F_{4}}{F_{4}}$
- $dT=T_{n}-T_{n-1}$

The variables in the equations are defined below:

- $S(T)$: the change in C_50_ as a function of temperature
- $F_{T}$: the C_50_ determined at temperature T
- $F_{4}$: the C_50_ determined at 4 °C
- $dT$: the difference between two adjacent temperature measurements


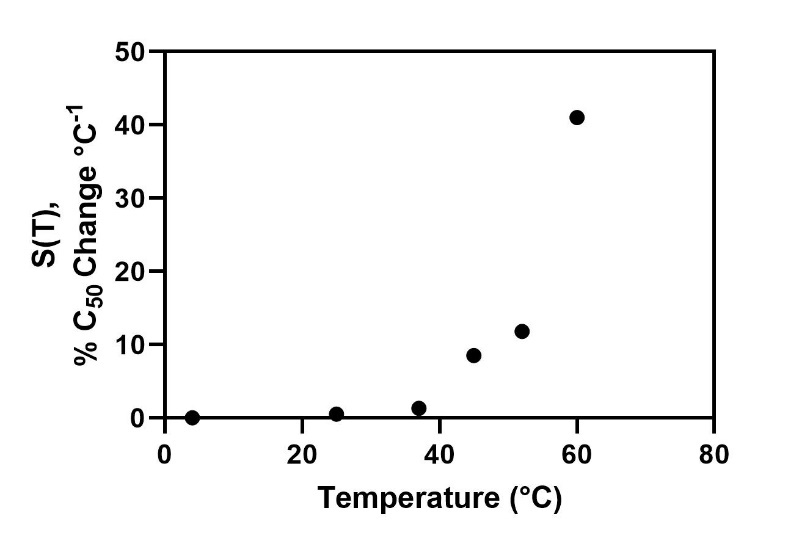


**Figure S46.** Change in the RR C_50_ as a function of temperature.


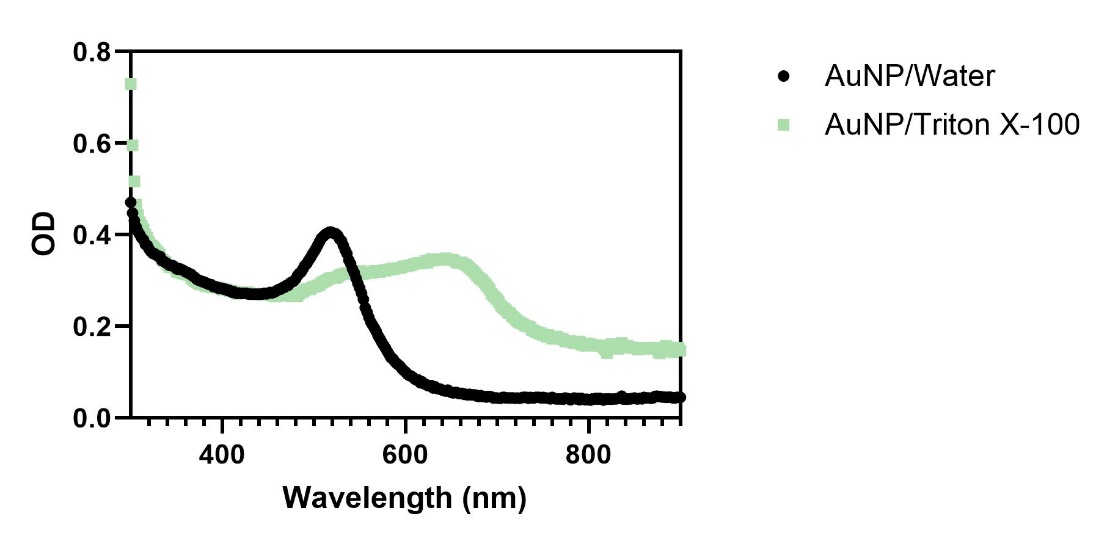


**Figure S47.** UV-vis spectra indicating the aggregation of the AuNPs in Triton X-100.

**
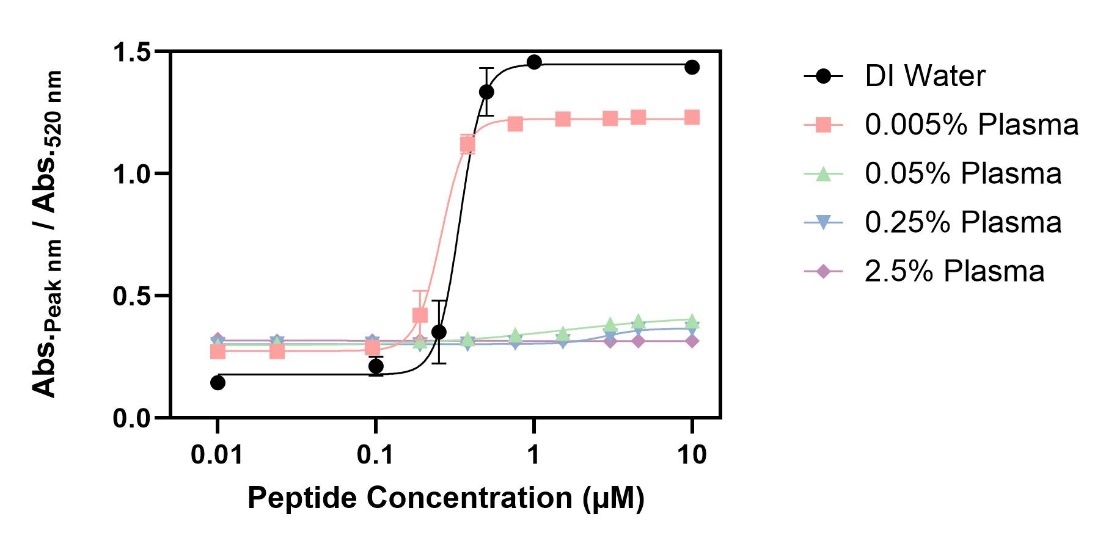
**

**Figure S48.** Dose-response curves indicating that the RR peptide could only induce the absorbance shift when the plasma was diluted to below 0.05%. The error bars represent the standard deviation of three replicates.


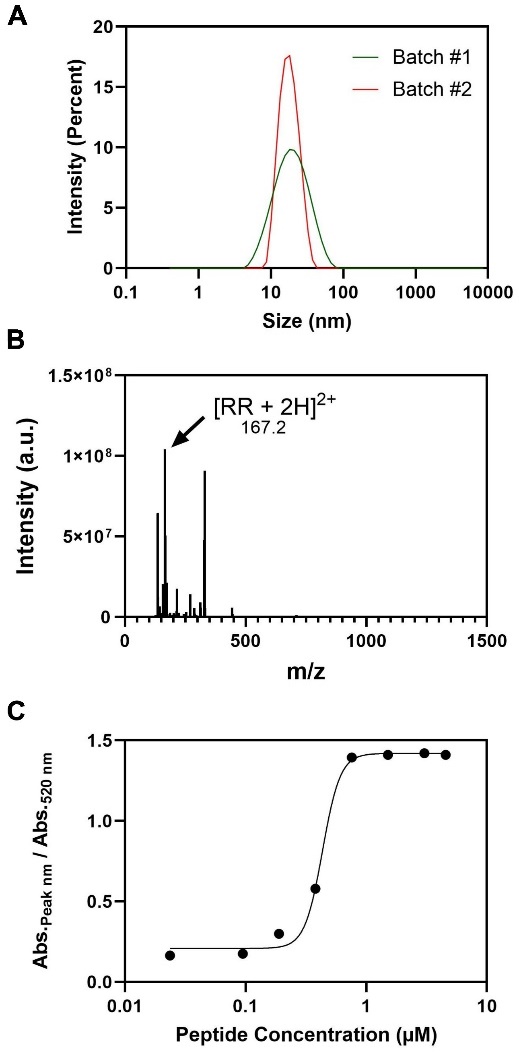


**Figure S49.** (**A**) DLS measurements comparing the sizes of AuNPs in two different synthesis batches. The sizes of the AuNPs were comparable with an average size of 18 nm for Batch #1 and 20 nm for Batch #2. (**B**) ESI-MS mass spectra of another synthesized batch of RR. (**C**) Dose-response curve of the aggregation of the AuNPs with RR from the second synthesis. The C_50_ value of 0.40 µM was comparable to the C_50_ of 0.34 µM from the first batch.


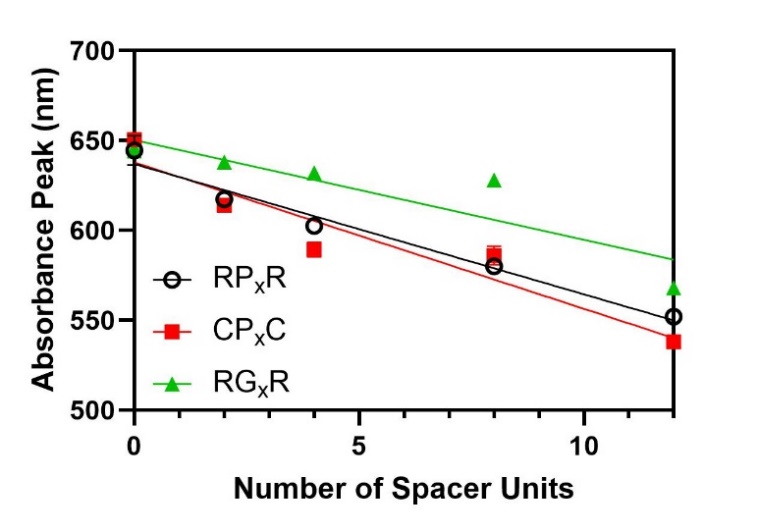


**Figure S50.** Plot illustrating diminished redshift upon AuNP aggregation with an increasing number of spacer units for the peptides RP_x_R and CP_x_C, and RG_x_R.


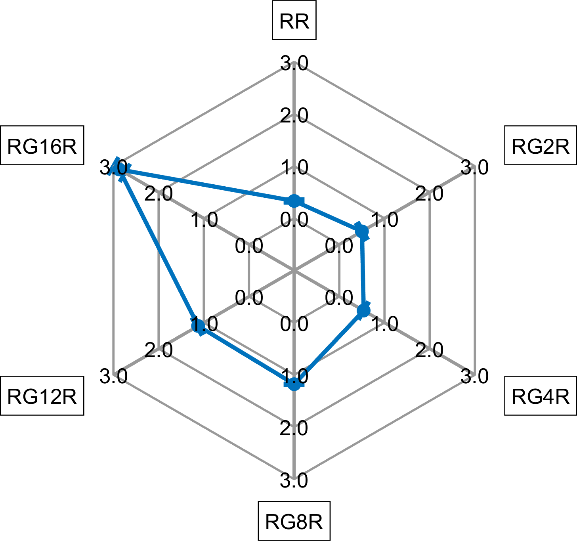


**Figure S51.** Radarplot comparing the C_50_ values of RR, RG_2_R, RG_4_R, RG_8_R, RG_12_R, and RG_16_R peptides. The error bars represent the standard deviation of three replicates.


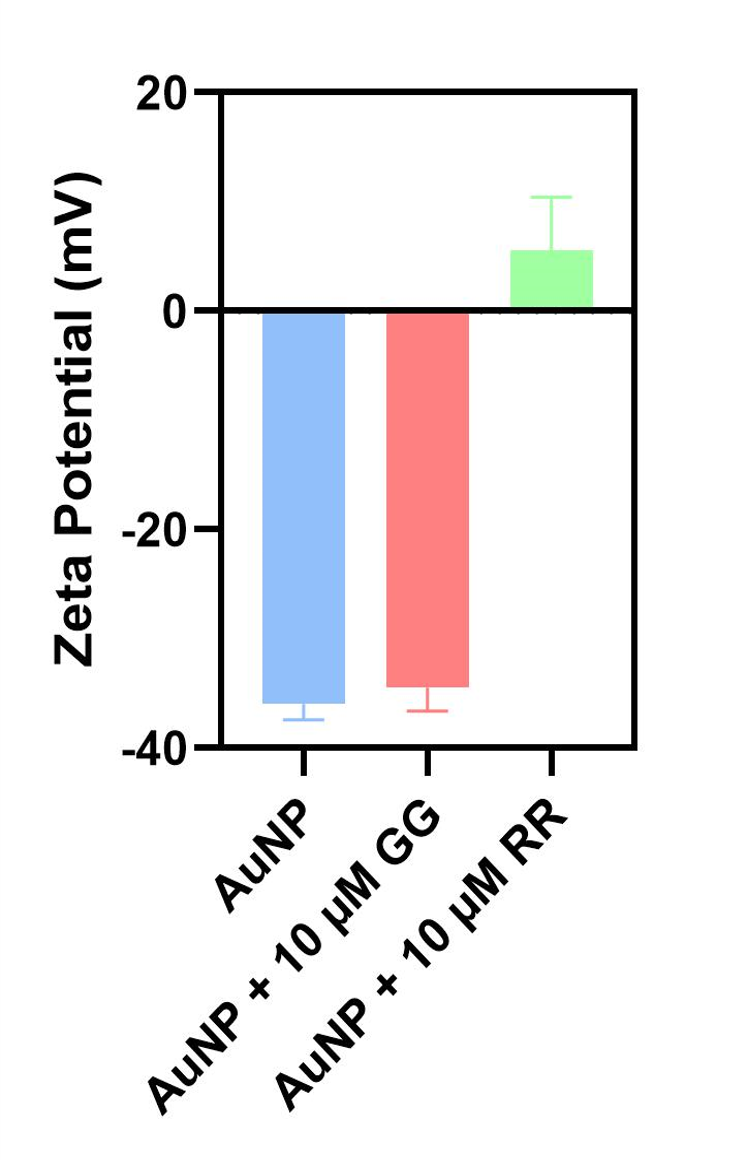


**Figure S52.** Plot of zeta potential measurements demonstrating the change in surface charge upon aggregation with the RR peptide. The error bars represent the standard deviation of three replicates.

VII. References

1. **PepDraw:** https://pepdraw.com/

2. **Protein Property Calculator**: http://biotools.nu-bic.northwestern.edu/proteincalc.html

3. **ESM Metagenomic Atlas:** https://esmatlas.com/

4. **ChimeraX**: https://www.rbvi.ucsf.edu/chimerax/

Molecular graphics and analyses performed with UCSF ChimeraX, developed by the Resource for Biocomputing, Visualization, and Informatics at the University of California, San Francisco, with support from National Institutes of Health R01-GM129325 and the Office of Cyber Infrastructure and Computational Biology, National Institute of Allergy and Infectious Diseases.

5. **BACHEM:** https://www.bachem.com/knowledge-center/peptide-calculator/

6. **Fractal Dimension Calculations:** https://github.com/pranurs/fractal-dimensions

Fractal dimensions were calculated using the getfractaldim() function.

7. **PEP-FOLD3**: https://bioserv.rpbs.univ-paris-diderot.fr/services/PEP-FOLD3/

8. **Radarplot**: https://www.mathworks.com/matlabcentral/fileexchange/59561-spider_plot

Radarplot was created using the spider_plot function.

9. **Surface Area Calculations**: Retout, M.; Mantri, Y.; Jin, Z.; Zhou, J.; Noël, G.; Donovan, B.; Yim, W.; Jokerst, J. V. Peptide-Induced Fractal Assembly of Silver Nanoparticles for Visual Detection of Disease Biomarkers. *ACS Nano* **2022**, *16* (4), 6165–6175. DOI: 10.1021/acsnano.1c11643.

Methodology to estimate peptide surface area and surface area coverage of the nanoparticles was adapted from this reference.

10. **CREST**: https://crest-lab.github.io

Pracht, P.; Grimme, S.; Bannwarth, C.; Bohle, F.; Ehlert, S.; Feldmann, G.; Gorges, J.; Müller, M.; Neudecker, T.; Plett, C.; Spicher, S.; Steinbach, P.; Wesołowski, P.A.; Zeller, F.; CREST — A program for the exploration of low-energy molecular chemical space, *J. Chem. Phys.*, **2024**, *160*, 114110. DOI: 10.1063/5.0197592

11. **Q-Chem**: https://www.q-chem.com

Epifanovsky, E.; et al.; Software for the frontiers of quantum chemistry: An overview of developments in the Q-Chem 5 package, *J. Chem. Phys.*, **2021**, *155*, 084801. DOI: 10.1063/5.0055522

12. **VMD:** https://www.ks.uiuc.edu/Research/vmd/

Humphrey, W.; Dalke, A.; Schulten, K., VMD - Visual Molecular Dynamics, *J. Molec. Graphics*, **1996**, 14, 33-38.
